# Impact of Soy Isoflavones on the Epigenome in Cancer Prevention

**DOI:** 10.3390/nu6104218

**Published:** 2014-10-15

**Authors:** Maria Pudenz, Kevin Roth, Clarissa Gerhauser

**Affiliations:** Division Epigenomics and Cancer Risk Factors, German Cancer Research Center (DKFZ), Im Neuenheimer Feld 280, 69120 Heidelberg, Germany; E-Mails: m.pudenz@dkfz.de (M.P.); k.roth@dkfz.de (K.R.)

**Keywords:** soy, isoflavones, DNA methylation, histone modifications, chromatin, miRNAs, lncRNAs, phytoestrogens, hormone-related tumors, epigenetic, chemoprevention

## Abstract

Isoflavones (IF) such as genistein are cancer preventive phytochemicals found in soy and other legumes. Epidemiological studies point to a reduced risk for hormone‑dependent cancers in populations following a typical Asian diet rich in soy products. IF act as phytoestrogens and prevent tumorigenesis in rodent models by a broad spectrum of bioactivities. During the past 10 years, IF were shown to target all major epigenetic mechanisms regulating gene expression, including DNA methylation, histone modifications controlling chromatin accessibility, and non-coding RNAs. These effects have been suggested to contribute to cancer preventive potential in *in vitro* and *in vivo* studies, affecting several key processes such as DNA repair, cell signaling cascades including Wnt-signaling, induction of apoptosis, cell cycle progression, cell proliferation, migration and invasion, epithelial-mesenchymal transition (EMT), metastasis formation and development of drug-resistance. We here summarize the state-of-the-art of IF affecting the epigenome in major hormone-dependent, urogenital, and gastrointestinal tumor types and in *in vivo* studies on anti-cancer treatment or developmental aspects, and short-term intervention studies in adults. These data, while often requiring replication, suggest that epigenetic gene regulation represents an important novel target of IF and should be taken into consideration when evaluating the cancer preventive potential of IF in humans.

## 1. Introduction

### 1.1. Isoflavones as Cancer Preventive Agents

Diet plays an important role in daily life. People nowadays consider nutrition not just as nourishment, but are more conscious about food and define their lifestyle by what they eat. Also, there is growing public interest in achieving and sustaining good health through healthy eating.

**Figure 1:**
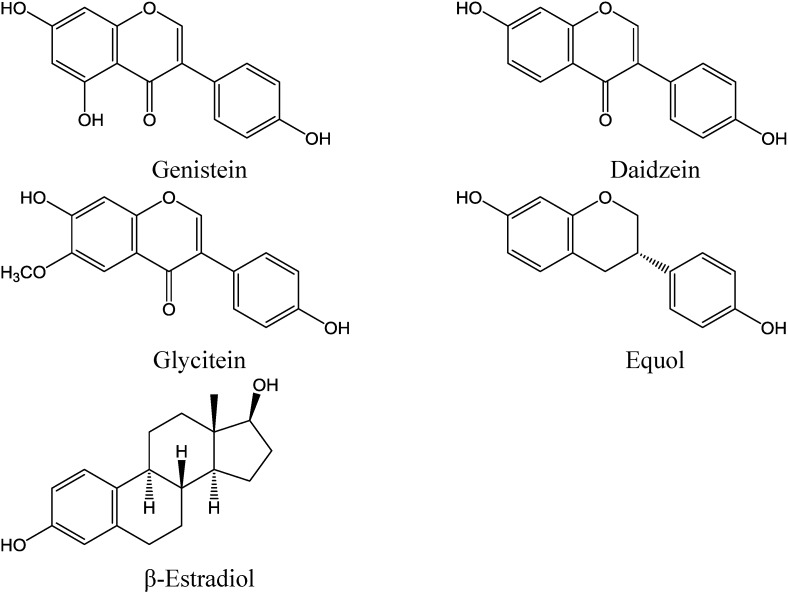
Chemical structures of soy isoflavones (IF) genistein (GEN), daidzein (DAI), glycitein (GLY), and the microbial daidzein metabolite equol in comparison with β-estradiol (E2).

The World Cancer Report 2014 states that intake of fruit, vegetables and phytochemicals from distinct botanical subgroups may contribute to a reduced risk of developing specific cancer subtypes [1]. Consequently, one of the recent dietary recommendations of the World Cancer Research Fund (WCRF) is to increase consumption of plant foods, particularly whole grains, nuts and legumes [2]. Legumes, lentils and chickpeas are a good dietary source of isoflavones (IF), a class of plant estrogens (phytoestrogens) predominantly found as glycoside conjugates. The most prevalent dietary IF including genistein (GEN), daidzein (DAI) and glycitein (GLY) (chemical structures in Figure 1) occur in nutritionally relevant amounts in soybeans and soy-based foodstuffs (up to 150 mg/100 g) [3]. Daily dietary intake of IF varies drastically between Western and Asian Countries. As fermented soy products like Tofu, Tempeh, Miso and soy sauce are part of the regular diet in many Asian countries, dietary intake of IF has been estimated from 15 mg/day in China [4] and 60 mg/day in Singapore [5] up to 200 mg/day in Japan [6]. In contrast, less than 3 mg/day IF are ingested in Western societies [7], but these numbers are higher for vegetarian- (12 mg/day) and vegan-based nutrition (70 mg/day) [8,9]. In addition to differences in uptake, variability in the gut microbiota composition that may influence IF bioavailability, as well as gene polymorphisms may explain discrepancies and individual variability observed in clinical studies investigating biological effects of IF [10].

The incidence of certain hormone-dependent cancer types, such as breast and prostate cancer, is lower in Asian countries than in Western Europe (age-standardized number of new cases per 100,000 inhabitants in 2012: breast cancer 29.1 *vs*. 96, prostate cancer 9.4 *vs*. 95) [11]. In addition, migration studies indicate that Japanese and Chinese American women had a 60% higher risk to develop breast cancer if they were born in Western countries compared to those born in the East. Women with grandparents also born in the West had an additional 50% higher risk [12]. Lifestyle and exposure to IF during development and early life thus may affect the etiology of certain cancers [13,14], and timing of exposure to IF seems to be a critical factor [15]. Especially multi-generational or pre-pubertal exposure was shown to modulate mammary gland morphology, resulting in anti-tumorigenic activity [16]. Since many processes during early development are regulated by epigenetic mechanisms, it is tempting to speculate that early epigenetic reprogramming of the mammary gland is targeted by IF, affecting normal cell growth and susceptibility to breast cancer [17].

Numerous transciptomic, proteomic and metabolomic studies have contributed to describe the complexity of biological effects of IF, but available data are partly inconsistent (reviewed in [10]). Due to structural similarities to endogenous 17β-estradiol (E2) (Figure 1), IF are able to selectively bind to estrogen receptors (ER) with distinct affinities, modulating the recruitment of co-repressors and co-activators and thus affecting ER-signaling [18]. GEN shows affinity to both ER-α and ER-β, since its hydroxyl groups are appropriately positioned to interact with the amino acids in the ER binding pocket. As amino acid residues vary between the ER subtypes, its binding capacity is higher for ER-β than for ER-α [19].

In addition to phytoestrogenic activity, IF affect a broad spectrum of mechanisms contributing to cancer preventive potential [10]. They exhibit antioxidant activity *in vitro* by scavenging free radicals and regulation of enzymes involved in antioxidative events, thus preventing cells from oxidative stress. IF also affect synthesis and metabolism of endogenous steroids, e.g., by inhibiting aromatase, a key enzyme involved in the conversion of testosterone to estrogens. Modulation of xenobiotic metabolism by inhibition of certain phase I enzymes (e.g., CYP1A1, 1A2) and concomitant induction of phase II enzymes (e.g., GSTs, NQO1, UDPGTs) by IF results in enhanced carcinogen inactivation and detoxification in both *in vitro* and *in vivo* studies. IF inhibit cell proliferation by regulating cyclin-dependent kinases (CDKs) or their inhibitors (e.g., p21, p16) and promote cell cycle arrest in G_2_/M. By inducing pro-apoptotic proteins from the BCL2 family, IF treatment was shown to induce apoptosis in variety of studies in cell culture, but also *in vivo*. IF further inhibit angiogenesis by down-regulating vascular endothelial growth factor (VEGF). Reduced expression of matrix metalloproteinases (MMPs) by IF blocks cell invasiveness and metastasis *in vitro* and *in vivo*. A number of studies highlight that IF are involved in the modulation of signaling pathways such as epidermal growth factor (EGF) and insulin-like growth factor (IGF-1) signaling, promoting cell differentiation over growth factor-stimulated proliferation and progression. Further, up-regulation of PTEN and inhibition of phosphorylation of IκB and ERK1/2 abrogated Akt and NFκB signaling *in vitro* and *in vivo*. These pathways are known to be involved in sustained cell proliferation and cell survival.

### 1.2. Epigenetic Mechanisms as Targets in Cancer Prevention

**Figure 2:**
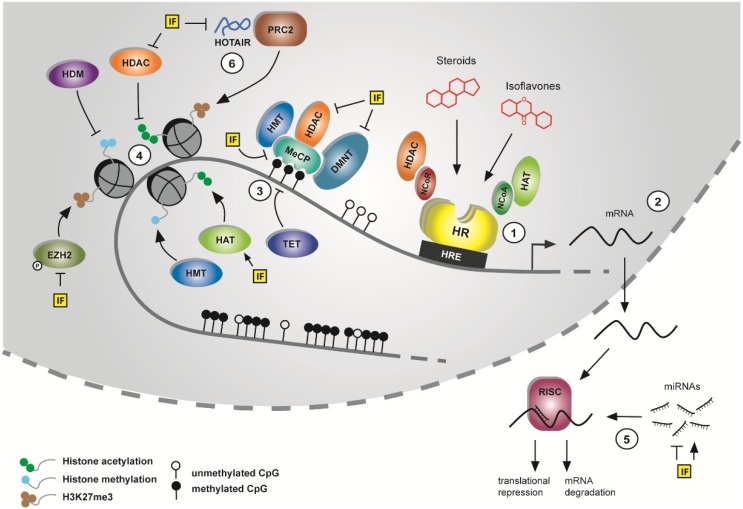
Overview of the impact of soy IF on the epigenome.

Notes: see text for abbreviations and details.

Epigenetic changes are heritable, but potentially reversible alterations in gene expression that do not result from changes in the DNA sequence. DNA methylation, histone tail modifications, nucleosome positioning and noncoding RNAs have the ability to establish altered gene expression as a consequence of endocrine signals or of environmental stimuli like diet or exposure to (phyto-)chemicals such as IF (overview in Figure 2) [20]. These mechanisms act coordinately to form an epigenetic landscape regulated by various enzymes establishing (writers), interpreting (readers), modifying (editors), or removing (erasers) epigenetic marks (reviewed in [21]).

A brief overview of the impact of IF on epigenetic mechanisms is given in Figure 2. (1) Binding of steroids such as E2, or IF to hormone receptors (HR) leads to receptor dimerization and, depending on cell type and context, recruitment of nuclear co-activator (NCoA) or co-repressor (NCoR) complexes associated with histone acetyltransferases (HAT) or histone deacetylases (HDACs), respectively, to hormone-responsive elements (HRE). (2) This interaction modulates the transcription of hormone-regulated genes, e.g., the estrogen-responsive HDAC6. (3) Accessibility of the transcription machinery to DNA is regulated by DNA methylation. In healthy tissue, CpG-dense promoter regions are normally unmethylated, whereas intergenic and especially repetitive sequences are generally highly methylated. Methyl-CpG-binding domain containing proteins (MeCP) bind to methylated DNA and recruit DNA methyltransferases (DNMTs), but also histone-modifying proteins such as HDACs and histone methyltransferases (HMTs) to fine-tune chromatin accessibility. Ten-eleven translocation (TET) proteins are involved in DNA demethylation and therefore contribute to reactivation of genes silenced by CpG methylation. IFs are able to inhibit MeCP expression as well as DNMT and HDAC expression or activity. (4) Dynamic positioning of activating (histone acetylation, H3K4me3) and repressive (H3K27me3) histone marks at gene promoter regions also strongly influences their accessibility. HDACs deacetylate histone tails while HATs catalyze the addition of acetyl groups. IF increase expression of HATs while having opposite effects on HDACs, leading overall to an increase of acetylated histones in gene promoter regions. Histone methylation is controlled by the interplay of histone methyltransferases (HMTs), such as enhancer of zeste homolog 2 (EZH2), and histone demethylases (HDMs) that add or remove methyl groups from histone tails, respectively. EZH2 is often overexpressed in cancer cells and leads to transcriptional repression by tri-methylation of histone 3 lysine 27 (H3K27me3). Genistein-mediated phosphorylation of EZH2 inhibited its activity and *promoted* benign uterine cancers in Eker rats [22]. (5) IF are also able to induce or repress levels of a multitude of tumor suppressive or oncogenic miRNAs by unknown mechanisms. Via the “RNA-induced silencing-complex” (RISC), these miRNAs lead to translational repression or mRNA degradation of their, often numerous, target genes and therefore provide another epigenetic mechanism by which IF can regulate pathways important for cancer development. (6) IF down-regulate, through miR-mediated mechanisms, the lncRNA HOTAIR, which is often overexpressed in cancer cells and, in interaction with the polycomb-repressive complex 2 (PRC2, containing EZH2, among other proteins) and the HDM lysine specific demethylase 1 (LSD1) couples H3K27 trimethylation and H3K4 demethylation for epigenetic silencing of a multitude of genes. Further details are explained below.

#### 1.2.1. DNA Methylation

Methylation at the C5 position of cytosines (5-mC) in the context of CG dinucleotides (CpG) is the most prevalent DNA-based epigenetic mark in the human genome and regulates active transcription of genes. The key players involved in establishing this mark are the DNA methyltransferase (DNMT) 1, responsible for maintaining the DNA methylation landscape after DNA replication during S-phase by preferentially converting hemimethylated DNA, as well as DNMT3a and 3b, which are involved in *de novo* methylation of fully unmethylated genomic regions. In healthy cells CpG-rich regions (CpG islands, CGIs) often found in gene promoters are usually unmethylated, allowing active transcription, with the exception of about 6%–8% of CGIs that are methylated to maintain tissue specific gene expression. On the other hand, interspaced CpG poor regions with repetitive genomic sequences are highly methylated to prevent these sites from active transcription [23]. Methyl-CpG-binding domain containing proteins (MeCP) recognize and specifically bind to methylated CpGs. They recruit co-repressor complexes associated with histone lysine methyltransferases (HMTs) and histone deacetylases (HDACs), or other chromatin remodelers that promote gene silencing. Methylation at transcription factor (TF) binding sites may protect from TF binding, thus modulating gene transcription. Passive loss of methylation during cell division by inhibiting or down-regulating DNMT1, or due to active demethylation by ten-eleven translocation (TET) proteins can lead to reactivation of previously silenced genes.

It is well known that epigenetic patterns undergo programmed alterations, best exemplified by a global demethylation in early pre-implantation, genome-wide *de novo* methylation after early embryogenesis, and age-related modifications possibly leading to cancer [24]. During carcinogenesis, loss of global methylation contributes to overall DNA hypomethylation particularly at repetitive sequences, facilitating genomic instability and chromosomal aberrations [25,26]. Region-specific hypomethylation at promoters of onco- and pro-metastatic genes has been reported as a mechanism of their activation [27,28,29,30,31,32]. Conversely, an increase in methylation (DNA hypermethylation) of promoter CGIs leads to transcriptional repression of tumor suppressor genes (TSGs) [33]. Since alterations in gene expression via epigenetic mechanisms are reversible, aberrant methylation has been identified as an attractive target for chemoprevention with dietary compounds.

#### 1.2.2. Histone Modifications

In addition to DNA methylation, chromatin accessibility and gene expression is controlled by various post-translational modifications of *N*-terminal histone tails, including acetylation, methylation, phosphorylation, ubiquitinylation, sumoylation, and ADP ribosylation [34,35]. Acetylation of histone tails by histone acetyltransferases (HATs) opens up the chromatin structure, allowing TFs to access the DNA. Consequently, proteins with HAT catalytic activity are often transcriptional co-activators, such as p300/CBP (CREB-binding protein) or PCAF (p300/CBP-associated factor). Histone acetylation is reversed by HDACs that remove histone acetyl groups by catalyzing their transfer to Coenzyme A (CoA), leading to chromatin condensation and transcriptional repression. Beside the currently known HDACs 1–11, structurally unrelated sirtuins (SIRTs) possess deacetylating activity, using NAD^+^ as a cofactor [36].

Histone methylation is another important epigenetic mark involved in regulation of chromatin structure. Histone methylation takes place at lysine and arginine residues. Histone lysine methylation has activating or repressive effects on gene expression, dependent on the lysine residue that is methylated (e.g., K4, K9, K27, K36, K79 in H3) and the methylation status (mono-, di-, or tri-methylation) [34]. Trimethylation at H3K4 (H3K4me3) is generally associated with active transcriptional start sites, whereas H3K9me2/me3 or H3K27me3 are found at transcriptionally repressed genomic regions [37]. So far, more than 50 HMTs have been identified in humans that transfer methyl groups from *S*-adenosyl-l-methionine (SAM) to lysine residues [37]. A prominent example is EZH2, a protein that is frequently up-regulated in cancer and catalyzes the methylation of H3K27 as part of the large multiprotein repressive complex PRC2 (polycomb repressive complex 2) [38]. Histone methylation marks are removed by histone lysine demethylases (HDMs), for example by Lysine Specific Demethylase 1 (LSD1) and the family of about 20 Jumonji domain-containing (JmjC) histone demethylases [34,37,39].

#### 1.2.3. Regulation of Gene Expression by Noncoding (micro) RNAs

MicroRNAs (miRNAs or miRs) are 20–23 nucleotide long single stranded RNA oligonucleotides that regulate gene expression. About 2800 human, 2000 mouse, and 830 rat miRs have been identified so far [40]. Biogenesis of miRNAs is a highly regulated multi-step process (reviewed in [41]). In the nucleus RNA polymerases II or III transcribe miR genes in primary miRs (pri-miRs). These pri-miRs are then cleaved into precursor hairpin miRs (pre-miR) by the Drosha-DGCR8 complex. Exportin-5-Ran-GTP exports pre-miRs to the cytosol, where Dicer in complex with TRBP (trans-activation response RNA-binding protein) cleaves the miR to its mature length. The guide strand of the mature miR is loaded into the “RNA-induced silencing-complex” (RISC) that targets mRNAs for degradation or translational repression. This process is however not universal for all miRs. Differences in biogenesis as well as miR editing provide a multitude of regulatory options to target miR regulation. In the case of perfect base-pairing with the target sequence, miR binding leads to mRNA degradation, whereas partial base-pairing blocks translation.

First evidence that miR down-regulation or deletion, particularly miR-15 and -16, plays a role in leukemogenesis was provided by Calin *et al.* in 2002 [42]. Since then, a multitude of miRs has been identified as important regulators in carcinogenesis and development of other diseases. MiRs can either function as tumor suppressor miRs or oncogenes (onco-miRs), and the expression profiles of various miRs vary in tumor compared to normal tissue. MiRNA profiles can serve as diagnostic tools and have been suggested to classify tumors more efficiently than mRNA-based methods. Profiling miRs can also be used as a prognostic tool. Several miRs have been linked to cancer prognosis and survival and may thus be used to guide treatment strategies (reviewed in [43]).

Regulatory RNAs do not only include miRs. The area of long non-coding RNAs (lncRNAs) is an emerging field in current cancer research. Gupta *et al.* discovered that the lncRNA HOTAIR shows increased expression in primary breast tumors and metastases and could be used to predict disease progression and development of metastasis [44]. Other lncRNAs have emerged as important regulators of cancer. SChLAP1 was discovered as a marker and regulator in prostate cancer [45], and higher levels of MALAT1 were found in non-small cell lung cancer [46]. All of these lncRNAs influence cell proliferation and migration, suggesting a role in tumor growth and metastasis, and could serve as diagnostic tools for cancer detection and progression.

## 2. Overview of Epigenetic Mechanisms Influenced by Soy Isoflavones according to Organ Type

This review summarizes current knowledge on how IF epigenetically regulate gene expression of TSGs and proto-oncogenes in various tissues and how these activities might affect the development and/or prevention of cancer (also reviewed in [47,48]).

### 2.1. Breast

With an incidence of 1.67 million newly diagnosed women and 521000 cancer death in 2012, breast cancer is the most common form of cancer affecting women worldwide [1,11,49]. In the US, it is estimated that there will be 232,670 new breast cancer cases in 2014, and 40,000 women will die of the disease [50]. The development of breast cancer is highly dependent on ovarian-associated hormones. Hormone-related risk factors like early onset of menarche, late menopause and elevated age of first pregnancy lead to prolonged exposure to elevated serum levels of sex hormones [51]. It has been reported that the incidence of hormone-related cancers varies about 10–20 fold between regions [52]. This might reflect insufficient screening possibilities in developing countries, but might also be affected by different environmental exposures and diets, as higher rates of hormone-dependent cancers are seen in populations following Western dietary habits generally low in fiber and high in sugar and fat. Women in Asian countries consuming a traditional diet low in fat, high in fiber and soy products show a reduced risk of developing breast cancer [53].

There is a multitude of preclinical *in vitro* and *in vivo* studies investigating the potential chemopreventive effects of IF [54]. Experimental investigations in rodents highlight the protective effects of GEN especially for chemically induced cancer. In contrast, rodent data investigating the impact of GEN on the growth of already existing tumors and in xenograft models in immunodeficient mice revealed a stimulatory effect on the proliferation of human breast cancer cells [55]. Recent systematic reviews on human interventional or observational data summarized that soy consumption may be associated with reduced risk of cancer incidence, recurrence and mortality. With respect to health risks, the authors concluded that although soy consumption appears to be generally safe, high doses above 100 mg/day cannot be recommended to breast cancer patients [56,57,58].

#### 2.1.1. IF Effects on Histone Modifications and DNA Methylation

GEN and other phytoestrogens are known to interact with ER-α and -β. After ligand binding, the ER dimer recruits nuclear co-activators such as SRC2 (steroid receptor co-activator 2), and the complex interacts with estrogen responsive elements (EREs) in promoter regions of estrogen-regulated genes (Figure 2). In contrast, anti-estrogen binding to the ER results in the recruitment of co-repressors [18]. Co-activators often have HAT activity, whereas co-repressors possess HDAC activity [59]. Studies on IF-mediated effects in breast (cancer) cells are summarized in Table 1.

In 2004, Hong *et al.* demonstrated in a test tube experiment with isolated ERs, co-activators, chromatin and [^3^H]-labeled acetyl-CoA as a substrate, that GEN, DAI, equol (a microbial metabolite of DAI), as well as AglyMax, a synthetic mix of 49.8% DAI, 14.9% GLY, and 6% GEN induced histone acetylation by increasing ER-mediated recruitment of SRC2 and the HAT p300. They observed more pronounced effects of all IF with ER-β than with ER-α [60].

Subsequent studies indicated that IF modulate epigenetic mechanisms including histone acetylation and DNA methylation also in cell culture and *in vivo*.

Long-term treatment of ER-positive MCF-7 breast cancer cells for 40–60 days with low dose GEN (10 nM) reduced basal histone H3 acetylation and desensitized the cells towards HDAC inhibitors trichostatin A (TSA) and apicidin. Cells were also less sensitive towards E2- or EGF-induced mitogenic stimulation and proliferation was reduced, suggesting that long-term GEN treatment might result in epigenetic changes that promote cancer preventive potential [61].

**Table 1:** Soy isoflavones targeting epigenetic mechanisms in breast (cancer) *in vitro* and *in vivo*.

Compounds and Concentration/Dose Tested	Treatment Time	Cell Lines—*In vivo* Models	Genes Regulated and Underlying Mechanisms	Methods Used—Comments	First Author, Year [Reference]
GEN, DAI, AglyMax, Equol at 12.5 nM–12.8 μM		*in vitro* with recombinant proteins	↑ ac. histones, via ER-α or ER-β-mediated activation of co-activator SRC2 and recruitment of HAT p300. Effect on ER-β >ER-α ER-α: E2 >equol >GEN >AglyMax >DAI ER-β: E2 >equol = GEN = AglyMax = DAI	*In vitro* HAT activity assay, co-incubation of purified chromatin and proteins, radioactive detection by SDS-PAGE	Hong, 2004 [60]
GEN 10 nM	40–60 days	MCF7 after long-term genistein treatment (LTGT)	↓ H3 expression and acetylation response after HDACi treatment ↓ growth response to HDACi TSA and apicidin	MTT assay Western blotting	Jawaid, 2010 [61]
GEN 5, 10, 25, 50 μM *In vivo*: GEN 250 mg/kg diet for 2 weeks prior to xenograft, alone and in comb. with TAM	1, 2, 3 day 6 weeks	MDA-MB-231 *in vitro* and as xenografts *in vivo*, C3(1) SV40 TAg mice	↑ ER-α expression (MDA-MB-231) ↑ sensitivity to E2 and TAM, ↑ PGR mRNA ↑ acH3, H3K9ac, acH4 at the ER-α promoter, especially in combination with TSA ↓ HDAC activity ↓ cell growth ↓ xenograft growth, esp. in comb with TAM ↓ tumor growth, ↓ PCNA staining, ↑ ER-α ↓ DNMT1 and HDAC1 mRNA	RT-qPCR Western blotting IHC	Li, 2013 [62]
GEN 2.5–400 μM *In vivo:* AIN-93G diet with GEN 250 mg/kg	3 days, 7 weeks	HMEC transformed with SV40, hTERT (SH) and Ha-Ras (SHR) *in vitro* and as xenograft *in vivo*	↑ apoptosis ↑ p21, p16 expression ↓ Bmi-1, c-Myc expression ↑ acH3 ↓ H3K27me3, H3K9me3 at p21 and p16 promoter ↑ H3K4me3, H3K9me3, H3K27me3 HMT activity (SHR cells) ↓ xenograft growth, ↓ tumor weight, ↓ PCNA ↑ p21 mRNA, ↓ c-Myc mRNA	MTT assay Flow cytometry Western blotting IHC ChIP-PCR HDAC activity assay (Active Motif) HMT activity assay (Epigentek) RT-qPCR	Li, 2013 [63]
GEN 18.5 μM DAI 78.5 μM Equol 12.8 μM	2 days	MCF-7 MDA-MB-231	↓ H3K27me3, ↓ H3K9me3, ↓ H3K4me3, ↑ H4K8ac, ↑ H3K4ac at selected gene promoters (EZH2, BRCA1, ER-α, ER-β, SRC3, p300) ↓ EZH2 (HMT) staining, ↑ p300 (HAT) expression	ChIP-PCR Immunocytochemistry	Dagdemir, 2013 [64]
GEN 3.125 μM MCF-7 (once in 1 week), MCF-10a (once/week for 2 weeks) MDA-MB-468 (3/week for 1 week)	1–2 weeks	MCF-7 MDA-MB-468 MCF-10a	↓ GSTP1 promoter methylation (MDA-MB-468) ↑ expression (MDA-MB-468) ↓ RARβ2 and HIN1 promoter methylation (MCF-10a)	MSP, RT-qPCR	King-Batoon, 2008 [65]
GEN 50 μM (MCF-10aT), GEN 100 μM (MCF-7)	0, 1, 2, 3 day	MCF-7 MCF-10aT (T24 Ha-Ras transformed)	↓ DNMT1, 3a, 3b, c-Myc protein expression ↑ E2F-1 protein expression ↓ methylation at E2F-1 binding site ↓ c-Myc- and ↑ E2F-1 promoter binding ↓ hTERT mRNA expression	BS, Luciferase Assay, RT-qPCR, Western blotting, ChIP-PCR, ChIP-BS	Li, 2009 [66]
GEN 18.5 μM DAI 78.5 μM	2 days	MCF-7 MDA-MB-231 MCF-10a	↓ BRCA1 (exon1) and BRCA2 (exon2) methylation, ↓ anti-5-mC and MeCP2 fluorescence signal ↑ expression BRCA1, BRCA2	MeDIP/PCR, IHC	Bosviel, 2012 [67]
Equol 2 μM	3 weeks (each for 2 days)	MCF-7 MDA-MB-231 MCF-10a	↓ BRCA1 and BRCA2 promoter methylation (not in MCF-10a), ↑ expression in the nuclei (BRCA1) and the cytoplasm (BRCA2)	qPCR-based quantitative analysis of methylated alleles (QAMA)	Bosviel, 2012 [68]
GEN 60, 100 μM	1, 2, 3 day	MCF-7 MDA-MB-231	↓ Cell viability and global DNA methylation ↓ DNMT activity, DNMT1 mRNA/protein expression ↓ promoter methylation and ↑ mRNA expression ATM, APC, PTEN, SERPINB5	SuperSense DNA Methylation Kit, EpiQuik DNMT Activity Assay Kit (Epigentek), MSP, RT-qPCR, Western blotting	Xie, 2014 [69]
*In vivo*					
Soy extract with 70.5% GEN, 27% DAI, 1.3% GLY Low dose: 37.2 mg/day High dose: 128.8 mg/day	28 days	American women, samples of mammary ductoscopy	↔ p16, RASSF1, RARβ2, ER, CCND2 methylation; RARβ2 methylation correlated with GEN serum levels (high dose group) and in low dose group for CCND2	qMS-PCR	Qin, 2009 [70]

Abbreviations: GEN: Genistein; DAI: Daidzein; GLY: Glycitein; AglyMax: synthetic mix of 49.8% DAI, 14.9% GLY, 6% GEN; HDACi: histone deacetylase inhibitor/inhibition; TSA: trichostatin A; PND: postnatal day; PNW: postnatal week; bw: body weight; SDS-PAGE: SDS-polyacrylamid gel electrophoresis; TAM: tamoxifen, a selective estrogen-receptor modulator; MTT: 3-(4,5-Dimethylthiazol-2-yl)-2,5-diphenyltetrazoliumbromid, a reagent for cell viability assays; RT-qPCR: reverse transcriptase-quantitative polymerase chain reaction; MSP: methylation-specific PRC; BS: bisulfite sequencing; ChIP: Chromatin Immunoprecipitation; IHC, immunohistochemistry; MeDIP, Methylated DNA Immunoprecipitation.

Epigenetic mechanisms contribute to reduced ER expression in ER-negative breast cancers [18]. Since these tumors do not respond to treatment with anti-estrogens such as tamoxifen (TAM), reactivation of ER expression might be an important strategy for the clinical management of the disease. In ER-negative MDA-MB-231 cells, GEN treatment led to increased histone acetylation at the ER-α promoter and increased ER-α expression. Also, the cells were re-sensitized to treatment with E2 and TAM, especially in combination with TSA. Accordingly, dietary GEN (250 mg/kg diet) in combination with TAM reduced tumor growth in a MDA-MB-231 xenograft model and in the C3(1) SV40 TAg transgenic mouse model for basal breast cancer [62].

In a subsequent study, Li *et al.* analyzed the potential of GEN to inhibit cell proliferation in human mammary epithelial cells (HMEC) transformed with SV40, hTERT (the catalytic subunit of human telomerase) and the oncogene Ha-Ras (SHR cell line). GEN induced apoptosis and up-regulated the expression of the cell cycle regulators p16 and p21, whereas the polycomb complex protein Bmi-1 and the proto-oncogene c-Myc were down-regulated. GEN treatment enhanced binding of acetylated histone H3 and reduced binding of the repressive histone marks H3K9me3 and H3K27me3 to p16 and p21 promoter regions. In a xenograft model with the SHR cell line, cell proliferation and tumor growth was strongly inhibited by dietary GEN (250 mg/kg diet) [63]. Similarly GEN, DAI, and equol reduced repressive histone marks H3K9me3 and H3K27me3 and enhanced activating histone marks H3K4ac and H4K8ac at the promoters of six selected genes, including BRCA1, ER-α and -β, the steroid receptor co-activator SRC3 interacting with ER, and the epigenetic regulators EZH2 (methylating H3K27) and p300 HAT (recruited by SRCs to EREs to open up the chromatin). The effect on H3K4me3 typically accumulating at transcription start sites was not conclusive [64].

Alterations in histone acetylation modulate chromatin accessibility and can indirectly affect DNA methylation. Recent investigations indicate that IF from soy are able to alter promoter methylation of TSGs in human breast cancer cell lines. Low GEN concentrations applied for 1–2 weeks were sufficient to reduce DNA methylation at the promoter of GSTP1, a Phase-II metabolizing enzyme commonly silenced in prostate cancer, with subsequent weak mRNA re-expression in ER-negative MDA-MB-468 but not in ER-positive MCF-7 cells. The promoters of the two TSGs RARβ2 (Retinoid acid receptor β2, a nuclear transcriptional regulator involved in regulation of cell growth and differentiation) and HIN1 (high in normal-1) were demethylated by the same low dose of GEN in the ER-negative cell line MCF-10a [65].

Li *et al.* analyzed effects of GEN on the expression of human telomerase reverse transcriptase (hTERT) in T24 Ha-Ras transformed MCF-10aT and MCF-7 cells [66]. hTERT is normally repressed in postnatal somatic cells, resulting in progressive shortening of telomeres as an important component of cellular aging. Reactivation of hTERT is a crucial event during cell transformation and promotes proliferation and survival potential independently of the telomere stabilization function. GEN treatment at relatively high doses (50–100 μM) increased expression of E2F-1, a repressor of hTERT transcription, resulting in enhanced binding to the hTERT core promoter and reduced hTERT mRNA expression. This was facilitated by reduced expression of DNMT 1, 3a and 3b proteins, which was suggested to reduce methylation at the E2F-1 binding site in the hTERT promoter [66].

The tumor suppressor genes BRCA1 and BRCA2 (Breast Cancer 1 and 2, early-onset) are involved in DNA repair mechanisms and are often mutated in breast cancer. Treatment with IF or equol at moderate doses resulted in demethylation of CpG sites in the promoter and/or exonic regions resulting in weak re-expression of the proteins in three breast cancer cell lines [67,68]. Immunohistochemical analyses revealed an overall decrease in 5-mC and MeCP2 protein expression after IF treatment.

In MCF-7 and MDA-MB-231 breast cancer cells, high concentrations of GEN (60, 100 μM) dose- and time-dependently reduced DNMT1 mRNA and protein expression and DNMT activity. This was accompanied by promoter demethylation and re-expression of four tumor suppressor genes, including ATM (ataxia telangiectasia mutated, an important cell cycle checkpoint kinase), APC (adenomatous polyposis coli, an antagonist of the Wnt-signaling pathway), the phosphatase PTEN, and SERPINB5 (mammary serine protease inhibitor encoding the Maspin protein) [69].

Notably, Qin *et al.* conducted a prospective, randomized, double-blind intervention trial with 34 healthy pre-menopausal American women receiving capsules with soy IF (low dose: 37 mg; high dose 128 mg daily, representative of a typical Asian diet) through one menstrual cycle. Breast tissue samples were obtained by mammary ductoscopy [70]. Serum GEN levels dose-dependently increased after the intervention. A nonsignificant trend toward lower complement C3 levels (a marker for estrogenic effects) was observed after IF intervention, indicating anti-estrogenic activity. Methylation of five candidate genes frequently silenced by methylation in breast and prostate cancer, including ER, RARβ2 and the cell cycle regulators p16, RASSF1 and CCND2 (cyclin D2) was analyzed in breast tissue. Methylation did not change in response to treatment. However, RARβ2 and CCND2 promoter methylation decreased with low and increased with high circulating post-treatment levels of GEN (RARβ2 below or above 600 ng/mL GEN; CCND2 below or above 200 ng/mL), suggesting a different mechanism of action for high *vs*. low GEN levels.

In conclusion, these studies provide good evidence that IF modulate the epigenome in breast cancer cell lines through histone modifying mechanisms, which might be due to a specific targeting of HATs such as p300 to E2-regulated genes via the recruitment of co-activators like SRCs through binding to EREs. IF-mediated effects on DNA methylation are weaker and less conclusive. This might partly be due to the fact that with one exception, so far only studies with selected candidate genes were performed, while a genome-wide view on DNA methylation changes after IF treatment is missing. A further limitation can be seen in the fact that so far, mainly cancer cell lines with an established epigenome were used to investigate IF effects, whereas the activity of IF might be more pronounced when analyzing their effects at specific time windows with active reprogramming during development.

#### 2.1.2. Ongoing Projects on Genome-Wide Methylome Profiling

To address some of the limitations of existing studies with IF on DNA methylation, we initiated several projects within the German Research Foundation (DGF)-funded IsoCross project to analyze genome-wide modulation of DNA methylation in rat mammary glands and mammary carcinogenesis, in cooperation with partners from the German Sports University in Cologne and the Technical University Dresden.

In the first study, we analyzed mammary glands of adult female Wistar rats treated with either an IF-depleted diet (IDD) or an IF-enriched diet (IRD) for 10 days during the hormonal decline after ovariectomy, with subsequent E2 exposure for three days. Genome-wide methylation data was generated by Methyl-CpG Immunoprecipitation (MCIp) [71]. Briefly, methylated DNA fragments were enriched by incubation with methyl-CpG-binding-domain protein 2 (MBD2) and fractionated by elution with a salt gradient, followed by next generation sequencing. Short term IF exposure had little impact on the methylation landscape of the normal mammary gland. In contrast, E2 treatment induced massive demethylation of the genome, especially at repetitive sequences, intronic and intergenic regions. These effects could in part be prevented when rats were exposed to IF prior to the E2 treatment, indicating reduced estrogen sensitivity (Pudenz *et al.*, in preparation).

In a follow-up study, we are currently using Reduced Representation Bisulfite Sequencing (RRBS) [72] to gain genome-wide quantitative DNA methylation data from long-term dietary IF intervention in August-Copenhagen-Irish (ACI) rats susceptible to E2-induced mammary carcinogenesis. Rats received either IDD or IRD starting *in utero* until postnatal day (PND) 240, allowing modulation of the epigenome during all critical developmental windows. At PND 45, the carcinogenic process was initiated by implantation of an E2-releasing tube. In this study we aim to analyze the influence of soy IF on DNA methylation during development and E2-induced mammary carcinogenesis. Results on DNA methylation will be integrated with information on gene expression to conclusively monitor the impact of DNA methylation changes on mammary carcinogenesis in this model, and to evaluate the modulating potential of IF.

### 2.2. Uterus and Ovaries

The uterus is the major female sex hormone-responsive reproductive organ of most mammals including humans. The lower end of the uterus opening to the vagina is designated as cervix, whereas the inner lining of the uterine cavity is called endometrium. Uterine tissue in pre-pubertal or ovariectomized rodents is very sensitive to estrogens; therefore, determination of proliferation or other modifications in uterine tissue is used to characterize the anti-/estrogenic potential of compounds. The ovaries are the most important organs for production of female gametes and female sex hormones including estrogens and progesterone. Studies on IF-mediated effects in the female reproductive system are summarized in Table 2.

#### 2.2.1. Uterus and Endometrium

Endometrial cancer is the most common type of uterine cancer. It typically arises after menopause and is related to estrogen exposure and obesity [2]. In 2012, worldwide there were 320000 new cancer cases and 76000 cancer death associated with endometrial cancer [1,11]. In the US, an estimated 52630 women will develop endometrial cancer in 2014, and 8590 will die of this disease [50].

**Table 2:** Soy isoflavones targeting epigenetic mechanisms in uterus and ovaries *in vitro* and *in vivo*.

Organ	Compounds and Concentration/Dose Tested	Incubation Time	Cell Lines—*In Vivo* Models	Genes Regulated and Underlying Mechanisms	Methods Used—Comments	First Author, Year [Reference]
**Uterus**	GEN 50 mg/kg bw by s.c. injection	PND 1–5	CD-1 mice (ovx and intact)	10 differentially methylated regions ovx: ↑ Nsbp1 promoter methylation and ↓ Nsbp1 expression intact: ↓ Nsbp1 promoter methylation and ↑ expression	Methylation sensitive restriction fingerprinting (MSRF), BS, RT-qPCR	Tang, 2008 [73]
	GEN *In vivo*: 60, 200 mg/kg diet *In vitro*: 10 μM	7 days	C57BL-6JJmsSlc mice, primary endometrial cells	↓ SF-1 promoter/1st exon methylation, most pronounced at luminal side of uteri ↑ SF-1 mRNA expression and downstream targets (Cyp11a1, StAR, Cyp17a1, Cyp19a1) ↔ proliferation	BS, High resolution melting assay (Roche), colony forming assay RT-qPCR	Matsukura, 2011 [74]
	GEN *In vivo*: 50 mg/kg bw by s.c. injection	PND 10–12	Eker rats	↑ PI3K/Akt signaling ↑ EZH2 phosphorylation ↓ H3K27me3 levels ↑ hypersensitivity of ER-responsive genes in neonatal uteri and adult myometrium ↑ uterine tumor incidence and multiplicity	RT-qPCR Western blotting	Greathouse, 2012 [22]
(Liver, Pancreas)	*In vivo*: 2% soy germ extract (Soylife) in the diet	Prenatal until PNW 6	C3H mice	↔ DNA methylation of skeletal α-actin, ER‑ɑ, c-fos; ↓ gender differences in methylation levels	semi-quantitative bisulfite PCR sequencing	Guerrero-Bosagna, 2008 [75]
**Cervix**	GEN 20 μM	6 days	SiHa	↓ RARβ2 promoter methylation, ↑ RARβ2 expression ↑ apoptosis	MSP	Jha, 2010 [76]
**Ovaries**	GEN 5 μM	2 days	UL-3A, UL-3B (from one patient during cancer progression)	↑ in UL-3A: miR-122a, -137, -196a, -204, -206, -217, -331, -449b, -454, -501, -515, -578 ↑ in UL-3B: miR-517c, -7 ↑ in UL-3A and 3B: miR-135, -765	miRNA microarray	Parker, 2009 [77]
	GEN 25, 50, 100, 200 μM	1–3 days	SKOV3	↓ miR-27a expression ↓ cell growth and migration ↑ SPRY2 expression	RT-qPCR Western blotting	Xu, 2013 [78]
(Uveal Melanoma)	GEN 10, 25, 50, 100, 200 μM; GEN 25, 50, 100 mg/kg bw i.p.	1.5–3 days, daily for 30 days	C918, C918 xenografts	↓ miR-27a expression ↑ ZBTB10 expression ↓ xenograft tumor growth	RT-qPCR	Sun, 2009 [79]

Abbreviations: ovx: ovariectomized; i.p.: intraperitoneal; also see footnotes Table 1.

Several studies on the developmental impact of soy IF addressed the question of whether phytoestrogen exposure might result in long-term changes in DNA methylation of uterine and other tissues. In an early study in 2008, Tang *et al.* used methylation sensitive restriction fingerprinting (MSRF) to mine changes in the uterine methylome in ovariectomized (ovx) or intact CD-1 mice that had received GEN (50 mg/kg body weight (bw)/day) for five days after birth [73]. In 6-month old ovx mice 10 genes were differentially methylated. Nsbp1 (nucleosome binding protein 1), a protein linked to open chromatin counteracting chromatin compaction, was selected for detailed analysis. In the absence of endogenous estrogens in ovx animals, GEN increased Nsbp1 promoter methylation and down-regulated mRNA expression in 6- and 18-month old mice. Intact mice showed opposite effects: Nsbp1 promoter methylation in general was higher in 6- and 18-month old intact mice than in ovx mice, and was rather reduced by GEN exposure, leading to up-regulation of mRNA expression (more pronounced in 6 month old mice). These results suggest that neonatal exposure to GEN is affecting uterine gene expression by reprogramming the epigenome. The response to GEN is highly dependent on adult ovarian steroid abundance.

Matsukura *et al.* investigated the effect of GEN on uterus and endometrium as estrogen-responsive and highly proliferative tissues [74]. Adult ovx mice were exposed for seven days to a low (60 mg/kg diet) or high dose of GEN (200 mg/kg diet), and methylation of SF-1 (steroidogenic factor 1, also known as Nr5a1, a transcription factor involved in steroidogenesis and often reactivated in human ectopic endometriosis) was evaluated in endometrial stromal cells. GEN treatment dose-dependently increased uterus wet weight, a marker for estrogenic properties, and decreased SF-1 methylation in the promoter and the 1st exon, mainly at the luminal side of the uteri. Demethylation of the SF-1 promoter was accompanied by a dose-dependent increase in SF-1 mRNA expression. As a consequence, the steroidogenic genes Cyp11a1, StAR (steroidogenic acute regulatory protein), Cyp17a1 and Cyp19a1 (aromatase) were induced. Primary endometrial cells harvested from uteri were treated with GEN (10 μM), but no significant effects on proliferation were observed. One clone with the highest proliferation rate showed significant SF-1 promoter demethylation, suggesting reactivation of SF-1 by GEN, followed by the induction of a steroidogenic cascade [74].

The influence of GEN exposure on uterine developmental reprogramming was also analyzed by Greathouse *et al.* in Eker rats that harbor a mutation in the Tsc2 (tuberous sclerosis complex 2) tumor suppressor gene and are predisposed to uterine leiomyomas. Leiomyomas are benign smooth muscle neoplasms arising from the uterine myometrium and can be induced in Eker rats by neonatal exposure to estrogen [22]. Both bisphenol A (BPA), a plasticizer with xenoestrogenic properties, and GEN (50 mg/kg bw) were applied on PND 10–12 and induced ER-signaling in neonatal uteri. However, only GEN activated PI3K (phosphatidylinositol 3-kinase)/AKT signaling, resulting in phosphorylation and subsequent inactivation of the HMT EZH2 and reduced levels of the repressive H3K27me3 histone mark. Also, only GEN developmentally reprogrammed estrogen-responsive genes to become hyperresponsive to E2. In contrast to BPA, exposure of neonatal Eker rats to GEN increased the incidence and multiplicity of leiomyomas. These data indicate that the differential response of the epigenetic regulator EZH2 to environmental estrogens plays an important role during developmental reprogramming and in the promotion of uterine tumorigenesis in this rat model.

A thematically related study that was interested in developmental effects of soy constituents, but focused on liver and pancreas as target tissues to analyze DNA methylation changes was performed in 2008 by Guerero-Bosagna *et al.* [75]. Simulating environmental exposure to high concentrations of dietary phytoestrogens, C3H mice were bred on a standard rodent diet, containing about 320 mg DAI and 154 mg GEN/kg diet, or the same diet supplemented with 2% of a soy-germ isoflavone (ISF) product (Soylife) enriched in all phytochemicals present in soy germ, including IF at a GEN:DAI:GLY ratio of 15:50:35. Final IF concentrations in the ISF diet were determined as about 0.39 g GEN and 1.61 g DAI/kg diet. In comparison to the control group, body size and weights at PND 42 were significantly lower in the ISF group, considering both males and females. Especially male pups at PND 42 were heavier in the control group than in the ISF group. Sexual maturity (vaginal opening) of female pups was advanced from PND 31.6 to PND 25.7 when bred on the ISF diet. With respect to DNA methylation, three genes were selected as surrogate markers for analyses: Acta1 (skeletal α-actin) known to be developmentally regulated, ER-α, and the proto-oncogene c-fos that harbors an ERE in its promoter region. Liver was chosen as a non-classical target organ for estrogens, and pancreas had been analyzed previously by Lyn-Cook *et al.* in a comparable study using the DAI metabolite equol (see pancreas) [80]. For Acta1, gender-specific differences in DNA methylation in the liver were suppressed in the ISF group, with weaker effects in pancreas. No gender- or diet-related differences in methylation at the ER-α promoter were detectable in liver, and c-fos was not methylated in both tissues. A drawback of this study might be seen in the fact that not only the ISF group, but also the control group was exposed to relatively high dietary levels of IF, thus complicating detection of a potential influence of IF on epigenetic reprogramming. In addition, only three genes were selected as surrogates for DNA methylation changes. Future studies on the developmental impact of IF and other phytoestrogens on the epigenome might benefit from genome-wide approaches and use of IF-free control diets for comparison.

#### 2.2.2. Cervix

Cervical cancer is the second most common cause of female specific cancers after breast cancer, accounting worldwide for about 528,000 new cases and 266,000 cancer deaths in 2012 [1,11]. Infections with human papilloma virus (HPV) have been identified as major risk factor, leading to the development of vaccines that are effective for cervical cancer prevention when applied before infection [81]. Food and nutrition do not seem to significantly modify the risk of cervical cancer, although the general nutritional status may affect a woman’s vulnerability to infection [2].

Expression of the retinoid receptor RARβ is reduced or silenced in many human cancers, including those of the head and neck, lung, esophagus, mammary gland, pancreas, and cervix, and epigenetic mechanisms including promoter methylation play a predominant role in its inactivation. GEN (20 μM for 6 days) reduced promoter methylation of RARβ2 in SiHa human cervical cancer cells, determined by semi-quantitative methylation-specific PCR (MSP), and increased RARβ2 mRNA levels [76].

#### 2.2.3. Ovaries

Ovarian cancer is more frequent in developed than in developing countries and has an overall poor prognosis. Globally, 239000 cases and 152000 deaths were reported in 2012 [1,11].

In 2009, Parker *et al.* [77] profiled miRNAs expression in response to GEN treatment in ovarian cancer cells. Two cell lines, UL-3A and UL-3B, were established from the same patient before and after cancer recurrence post treatment. In UL-3A, miR-122a, -137, -196a, -204, -206, -217, -331, -449b, -454, -501, -515 and -578 were up-regulated by GEN, while miR-517c and miR-7 showed higher expression in UL-3B cells. Two miRNAs, namely miR-135 and miR-765 were higher expressed in both cell lines upon GEN treatment [77]. These results suggested that GEN is able to induce expression of a multitude of miRNAs in ovarian and maybe other cancers, but did not provide hints on the mechanism underlying the observed changes.

Oncogenic miR-27a is involved in the development of resistance to doxorubicine in breast cancer [82]. In a study by Xu *et al.*, miR-27a was shown to be overexpressed in ovarian cancer tissues compared to benign tissue samples. Anti-miR-27a was able to reduce proliferation and migration, while a miR-27a mimic showed opposite effects. Treatment of ovarian cancer cells with GEN reduced miR-27a levels and consequently repressed cell proliferation and migration. The tumor suppressor SPRY2 (Sprouty 2) is a target for miR-27a and known to inhibit ERK signaling pathways. GEN treatment significantly increased SPRY2 expression, potentially through down-regulating miR-27a expression [78]. Similarly, GEN incubation of uveal melanoma cells with GEN dose-dependently reduced miR-27a expression *in vitro* [79]. *In vivo*, intraperitoneal injection of GEN (25–100 mg/kg bw/day) significantly reduced uveal melanoma cell xenograft growth in a dose-dependent manner. ZBTB10, a known target of miR-27a and putative repressor of the TF SP-1, showed higher expression in cells treated with 200 μM GEN, suggesting that reduced growth of GEN-treated cells and xenografts might partly be due to reduced miR-27a and therefore increased ZBTB10 expression [79].

### 2.3. Urogenital System

The urogenital or genitourinary system summarizes the organs of the reproductive and the urinary system. These are grouped together not only because of their anatomical proximity, but also due to their common embryological origin from the intermediate mesoderm [83]. Studies on IF-mediated effects in the urogenital system are summarized in Table 3.

#### 2.3.1. Prostate

Prostate cancer is the second most common form of cancer in men. Worldwide, more than 1.1 million men were diagnosed and 307000 died from the disease in 2012, with almost 70% of the cases occurring in more developed regions [1,11]. In the US, prostate cancer is the most common cancer type. It is estimated that 233000 new cases will be diagnosed and 29480 men will die of prostate cancer in 2014 [50]. In addition to hormone-related risk factors, other factors such as age, ethnicity and geographic location affect prostate cancer development. Incidence of prostate cancer varies up to 25-fold between geographic regions, with overall lowest rates in the Asian population [84]. Similar to breast cancer statistics, Asian migrants to the US develop an increased prostate cancer risk compared to their relatives still living in Eastern countries [85]. The relationship between soy intake and prostate cancer has been investigated in a variety of studies (review in [86]).

**Table 3:** Soy isoflavones targeting epigenetic mechanisms in the urogenital system *in vitro* and *in vivo*.

Organ	Compounds and Concentration/Dosetested	Incubation Time	Cell lines—*In Vivo* Models	Genes Regulated and Underlying Mechanisms	Methods Used—Comments	First Author, Year [Reference]
**Prostate**	GEN 1, 10, 25, 50 μM	3 days (applied every day)	LNCaP	↓ HDAC6 protein expression ↑ acetylation of HSP90 promotes dissociation and degradation of AR ↓ AR-mediated signaling, PSA levels	RT-qPCR Immunocytochemistry IP Western blotting HDAC6 siRNA	Basak, 2008 [87]
	GEN 10, 25 μM	4 days (applied every day)	LNCaP, DuPro	↑ p300, PCAF, CBP, HAT1 expression ↑ acH3, acH4, H3K4me2 at p21 and p16 promoter ↑ p21, p16^INK4a^ expression ↓ cyclin A2, B2, E2 expression ↑ cell cycle arrest, apoptosis (DuPro)	RT-PCR ChIP-PCR Western blotting flow cytometry	Majid, 2008 [88]
	GEN 1, 10, 25, 50 μM GEN 50 μM in comb. with TSA 300 nM for 1day	1, 2, 3 day	LNCaP, PC-3	↑ PTEN, CYLD, p53, FOXO3a expression ↓ Akt signaling ↓ SIRT1 activity/expression ↑ acH3K9 and ↓ H3K9me2 at PTEN, CYLD and FOXO3a promoter	RT-PCR Western blotting BS ChIP-PCR	Kikuno, 2008 [89]
	GEN (10, 25), 50 μM	3 days	LNCaP, PC-3, RWPE-1 prostate epithelial cells	↑ BTG3 expression ↓ BTG3 promoter methylation ↓ DNMT 1, 3a, 3b levels ↓ MBD2 binding activity ↑ HAT activity, ↔ HDAC activity ↑ acH3, acH4, H3K4me2, H3K4me3 ↔ H3K9me2, H3K9me3	RT-PCR BS EpiQuik DNMT, MBD2, HAT, HDAC activity kits (Epigentek) ChIP-PCR	Majid, 2010 [90]
	GEN *In vivo:* 300 mg/kg diet	2–4 weeks	C57BL/6J male mice	3/900 regions differentially methylated	BstUI/HpaII digestion; mouse differential methylation hybridization (mDMH)	Day, 2002 [91]
	GEN 10, 20 μM	6 days	LNCaP, PC-3	↓ RARβ2 promoter methylation, ↑ RARβ2 mRNA expression	MSP, RT-qPCR	Fang, 2005 [92]
	GEN 40 μM, DAI 110 μM	2 days	LNCaP, DU 145, PC-3	↓ GSTP1, EPHB2 (PC-3) and RASSF1A (PC-3 and LNCaP) promoter methylation ↔ BRCA1 GEN >DAI ↑ nuclear protein expression of GSTP1 and EPHB2 (DU 145)	MSP, IHC	Vardi, 2010 [93]
	GEN 40 μM DAI 110μM	2 days	DU 145 PC-3	↓ BRCA1, GSTP1, EPB2 promoter methylation (GEN), ↑ BRCA1, GSTP1, EPB2 expression (PC-3) ↓ BRCA1, GSTP1 expression (DU 145)	Methylation Profiler Methylation Kit (Qiagen), Western blotting	Adjakly, 2011 [94]
	GEN 20 μM	6 days	DU 145 PC-3 LNCaP, ARCaP-E, ARCaP-M	↔ APC, SOX7, SFRP1, WIF1 promoter methylation ↔ genome-wide DNA methylation (27 k) ↑ H3K9ac at APC, SOX7, SFRP1, SFRP2, DKK, WIF1 promoter, ↑ HAT1 expression ↑ SOX7, SFRP1 mRNA expression ↓ proliferation, ↑ apoptosis in comb. with vorinostat (HDAC inhibitor) ↑ DNA repair genes BRCA1, BARD1, RAD23B, XRCC2 ↓ expression of BIRC7, SLUG, HES1, TGFB1I1	MSP, Illumina 27 k, BS ChIP-PCR Western blotting RT-qPCR Whole genome expression profiling	Phillip, 2012 [95]
	GEN 40 μM DAI 100 μM	2 days	DU 145 PC-3 LNCaP	↓ miR-125a, -125b, -15b, -320, -155, -208b, -211, -376a, -411, -520g, -542-5p ↑ miR-548b, -15a	RT-qPCR	Rabiau, 2011 [96]
	GEN 25 μM (in comb. with 5 μM decitabine and TSA)	4 days	DU 145 PC-3 PWR-1E	↑ miR-145 expression ↑ TNFSF10 expression ↑ apoptosis and ↓ CDK6 in miR-145-overexpressing cells	RT-qPCR mRNA microarray Western blotting	Zaman, 2010 [97]
	GEN 25, 50 μM	4 days	LNCaP PC-3	↑ miR-1296 levels ↓ expression of MCM genes ↓ CDK2, CDK7, CDT1 expression ↓ cells in S-phase	RT-qPCR Western blotting	Majid, 2010 [98]
	GEN 50 μM	4 days	PC-3 LNCaP DU 145 patient samples	↓ miR-221, miR-222 levels ↑ ARHI expression ↓ proliferation, ↑apoptosis in cells overexpressing ARHI	RT-qPCR Western blotting	Chen, 2011 [99]
	GEN 25 μM	4 days	PC-3, DU 145, RWPE-1	↓ miR-151a-5p ↑ SOX17, ARHGDIA in miR-151a-5p precursor transfected cells	RT-qPCR Western blotting	Chiyomaru, 2012 [100]
	G2535 (equivalent to 20 μM GEN)	5 days	LNCaP, PC-3, VCap, V4-2B, ARCaP-M	↓ methylation at miR-29a and miR-1256 promoter ↑ miR-29a and miR-1256 ↓ TRIM68 and PGK-1 ↓ cell growth and invasion	Illumina 450k array MSP microarray Luc-Pair miR Luciferase assay, Western blotting	Li, 2012 [101]
	GEN 25, 50 μM	4 days	PC-3 DU 145 RWPE‑1 patient samples, xenografts	↑ miR-574-3p ↓ RAC1, EGFR, p300 in miR-574-3p precursor transfected cells ↓ proliferation, ↑apoptosis in cells overexpressing miR-574-3p	RT-qPCR Western blotting 5’UTR luciferase reporter assay	Chiyomaru, 2013 [102]
	GEN 25 μM	4 days	PC-3 DU 145 Xenografts	↑ miR-34a ↓ HOTAIR expression ↓ proliferation, ↑apoptosis	microarray RT-qPCR Dual-luciferase reporter assay	Chiyomaru, 2013 [103]
	GEN 25 μM	4 days	PC-3 DU 145 RWPE-1	↓ miR-1260b expression ↑ SFRP1, Smad4 expression ↓ CpG methylation at SFRP1 promoter ↓ H3K9me2, H3K9me3, H3K27me3 at SFRP1 and Smad4 genes ↓ proliferation, ↓invasion, ↓migration ↑ apoptosis	microarray BS, ChIP-PCR, RT-qPCR Western blotting	Hirata, 2014 [104]
**Kidney**	GEN 10, 25, 50 μM	3 days	A498, ACHN, HEK-293, HK-2 non-malignant immortalized renal cells	↓ cell growth, cell cycle progression ↑ BTG3 expression ↓ BTG3 promoter methylation ↓ DNMT activity, DNMT 3b levels ↓ MBD2 binding activity ↑ HAT activity, ↓ HDAC activity ↑ acH3, acH4, H3K4me2, H3K4me3 ↔ H3K9me2, H3K9me3	flow cytometry MTT assay RT-qPCR BS EpiQuik DNMT, MBD2, HAT, HDAC activity kits (Epigentek) ChIP-PCR	Majid, 2009 [105]
	GEN 25 μM	4 days	A-498 *in vitro* and *in vivo*	↓ miR-21 expression ↓ xenograft growth (pretreated with 25 μM GEN for 4 days)	RT-qPCR	Zaman, 2012 [106]
	GEN 25, 50 μM	4 days	A-498, ACHN, Caki-1, -2	↓ miR-23b-3p ↑ PTEN in miR-23b-3p knockdown cells ↓ proliferation, ↓ invasion in miR-23b-3p knockdown cells	RT-qPCR Western blotting	Zaman, 2012 [107]
	GEN 25 μM	4 days	RCC patient samples, A-498, 786-O, Caki-2	↓ miR-1260b expression ↓ Wnt-signaling ↑ viability, ↑ invasion ↓ apoptosis in miR-1260b transfected cells	microarray RT-qPCR TOPflash luciferase assay	Hirata, 2013 [108]
	GEN 25 μM	4 days	786-O ACHN HK-2	↑ miR-141 levels ↓ HOTAIR expression ↓ ABL2 expression ↑ PCDH10 in pre-miR-141 transfected cells ↑ Snail in anti-miR-141 transfected cells ↓ proliferation in HOTAIR knockdown cells ↑ proliferation in pre-miR-141 expressing cells	RT-qPCR Luciferase reporter assay	Chiyomaru, 2014 [109]

Abbreviations: RCC: renal cell carcinoma; also see footnotes Table 1.

Development of prostate cancer is accompanied by accumulation of genetic and epigenetic aberrations and deletion or silencing of TSGs, often in an androgen-dependent manner [110]. Androgen-mediated androgen receptor (AR) signaling provides the most important growth stimulus in hormone-dependent prostate cancer. Interestingly, GEN treatment reduced AR-mediated signaling and secretion of prostate-specific antigen (PSA) in AR-positive LNCaP cells. Mechanistically, anti-estrogenic activity of GEN lowered HDAC6 expression levels. This led to increased acetylation of the HDAC6 target heat shock protein 90 (HSP90) that functions as an AR chaperone, thereby dissociating the interaction between HSP90 and AR. Consequently, AR levels were reduced through enhanced proteasomal degradation. The effects of GEN-mediated HDAC6 down-regulation on AR-signaling were mimicked by HDAC6 siRNA, thus confirming the importance of this mechanism for prostate cancer preventive potential of GEN [87].

In addition to transcriptional repression of HDAC6, GEN induced expression of several HATs, including p300, PCAF, CBP, and HAT1 in LNCaP and DuPro prostate cancer cell lines and the normal epithelial prostate cell line RWPE-1. This resulted in histones H3 and H4 hyperacetylation, enhanced binding of acetylated H3 and H4 to the p21 and p16 promoters, induction of their expression, cell cycle arrest and apoptosis [88].

The TSG PTEN is inactivated in prostate cancer by epigenetic mechanisms, including miRNA silencing and lncRNA-mediated mechanisms [111]. PTEN regulates the PI3K/AKT pathway that promotes cell proliferation, growth and motility [112]. GEN treatment reduced AKT signaling in LNCaP and PC-3 cells, accompanied by up-regulation of PTEN and of the downstream mediators p53 and FOXO3a. GEN also induced the endogenous NF-κB inhibitor CYLD, resulting in decreased constitutive NF-κB activity. Further mechanistic analyses indicated that re-expression of PTEN, p53, CYLD and FOXO3a was associated with increased H3K9 acetylation at their promoters. In this study, increased histone acetylation was linked to reduced expression and nuclear localization of the histone deacetylase SIRT1. These findings underline the importance of epigenetic mechanisms for the GEN-mediated inhibition of PTEN/AKT and NF-κB signaling in prostate cancer [89].

Activation of HAT activity by GEN was involved in the re-expression of BTG3 (B-Cell translocation gene 3) in prostate cancer cell lines. The TSG BTG3 is a negative regulator of E2F-1 signaling and often silenced in prostate and other types of cancer by DNA hypermethylation. GEN reduced promoter methylation of BTG3 to levels observed in normal prostate epithelial cells. Decreased DNMT1, 3a and 3b activity and lower MBD2 (methyl-CpG binding domain protein 2) binding to the BTG3 promoter was accompanied by enhanced HAT activity. Elevated levels of acetylated H3 and H4 as well as activating H3K4me2 and H3K4me3 marks at the BTG3 promoter indicated increased transcription [90], and further support the link between modulation of histone modifications and the influence of GEN on gene expression.

In 2002, Day *et al.* were first to determine the effects of GEN on DNA methylation in healthy mice *in vivo* [91]. Male C57BL/6J mice received a casein-based diet supplemented with 300 mg GEN per kg for up to 4 weeks in a crossover design. DNA from liver and prostate was subjected to methylation-sensitive restriction enzyme digestion and subsequent hybridization to custom arrays. In the liver, no DNA methylation changes were observed. In prostate tissue, three regions out of 900 investigated were found to be differentially methylated after 4 weeks GEN treatment. Similar methylation changes in prostate tissue were also observed after 2 weeks intervention, independent of whether the animals had received the casein-based or the GEN-supplemented diet first. The results suggest that dietary exposure to GEN is able to affect DNA methylation in mice and results in few but stable alterations in prostate tissue.

In a study of Fang *et al.*, GEN dose-dependently reduced promoter methylation of the RARβ2 TSG and induced mRNA re-expression in prostate cancer cell lines [92]. Vardi *et al.* investigated the demethylating properties of IF on EPHB2, a receptor tyrosine kinase commonly mutated in prostate cancer, RASSF1A (Ras Association Domain family 1, isoform A), a TSG involved in cell cycle arrest and frequently hypermethylated in prostate cancer, as well as on GSTP1 and BRCA1. Methylation at the GSTP1, EPHB2 and RASSF1A promoters were reduced after treatment with GEN, DAI and the DNMT inhibitor decitabine alone or in combination, whereas BRCA1 was not affected. GEN was more effective than DAI. GSTP1 protein expression was weakly up-regulated in the nucleus, and elevated levels of EPHB2 were detected in the cytoplasm. RASSF1A protein levels were not induced, suggesting an additional level of regulation of expression [93].

In a subsequent study of this group, a methylation profiler kit was used to quantitatively determine DNA methylation changes. Methylation data from the previous study [93] were confirmed, and additionally BRCA1was found to be demethylated by IF treatment. Western blotting analysis indicated a weak to modest increase in protein expression for BRCA1, GSTP1 and EPHB2 in PC-3, but not in DU 145 cells (except for EPHB2) [94]. As effects of IF were similar to those obtained with decitabine, results suggest that soy IF are promising agents for the prevention or treatment of prostate cancer.

Phillip *et al.* [95] investigated the effect of IF alone or in combination with the HDAC inhibitor vorinostat on the epigenetic state of Wnt-inhibitory genes in several prostate cancer cell lines as well as in an epithelial-to-mesenchymal (EMT) transition model (ARCaP-E and ARCaP-M). Illumina 27 k technology was used to monitor genome-wide DNA methylation at 27000 CpG sites located in CGI associated with about 14000 genes. No significant changes were observed after IF treatment. Lack of demethylating activity of GEN was confirmed by alternative methods, including MSP for APC, SOX7, SFRP1 (secreted frizzled related protein 1) and WIF1 (Wnt-inhibitory factor 1), as well as by Bisulfite Sequencing (BS) for WIF1. Instead, GEN treatment increased HAT1 protein expression and enhanced H3K9 acetylation at APC, SOX7, SFRP1, SFRP2, DKK and WIF1 promoter regions which was accompanied by increased SOX7 and SFRP1 mRNA expression. GEN treatment reduced proliferation and induced apoptosis alone or in combination with vorinostat with a more than additive effect on cell death for ARCaP-E and ARCaP-M cells. The combination of GEN with the HDAC inhibitor was more effective than with the DNMT inhibitor decitabine. Using whole genome expression profiling, the GEN-vorinostat combination was shown to modulate expression of several chromatin regulators (HAT1, SIRT, CBP). BIRC3 encoding an inhibitor of apoptosis, TNFα, and the DNA repair-related genes BRCA1 and BARD1 were up-regulated, whereas BIRC7/livin, SLUG involved in EMT, HES1, a regulator of E2F TFs, and TGFB1I1, a co-activator of the androgen receptor, were down-regulated. These data indicate that GEN is able to affect cell survival and proliferation *via* multiple mechanisms involving chromatin modifications and histone acetylation. GEN can cooperate with vorinostat to induce cell death which was most pronounced in ARCaP-E cells representing early stage prostate cancer [95].

Several studies have recently addressed the question whether IF modulate gene expression in prostate cancer by miR-mediated mechanisms. One study investigated the expression of the lncRNA HOTAIR in response to GEN treatment.

In a comparative low density miRNA profiling study, GEN (40 μM) and DAI (110 μM) changed the expression profiles of several miRNAs in PC-3, DU 145, and LNCaP cell lines. While miR-15b, -125a, -125b, -155, -208b, -211, -320, -376a, -411, -520g and -542-5p were down-regulated after 48 h, miR-15a and miR-548b were up-regulated. The results suggest that IF are able to change the expression of a large number of miRNAs by hitherto unknown mechanisms and might therefore be able to influence several cellular pathways [96].

MiR-145 is a putative tumor supressor miR silenced by promoter methylation in prostate cancer [97,113,114]. GEN, in combination with decitabine, was able to re-express miR-145. Additional treatment with the HDAC inhibitor trichostation A (TSA) further up-regulated miR-145 expression, suggesting that histone acetylation is also involved in its regulation. RNA microarray analyses of PC-3 cells overexpressing miR-145 indicated that TNFSF10 (TNF-related apoptosis-inducing ligand TRAIL) was significantly up-regulated and might be an important target of miR-145 in prostate cancer [97].

The minichromosome maintenance (MCM) gene family is frequently up-regulated in various cancers, including prostate cancer. This gene family plays an essential role in DNA replication as MCM2-MCM7 complexes have helicase activity and assist in DNA replication. A study by Majid *et al.* [97] indicated that GEN treatment down-regulated MCM genes in cancer cell lines and significantly decreased the number of cells in S-phase; no effect on apoptosis was observed. GEN also significantly down-regulated the expression of CDK2, CDC7 and CDT1 required by the MCM2-MCM7 complex to be loaded onto chromatin. Mechanistically, GEN dose-dependently induced expression of miR-1296, which is down-regulated in prostate cancer samples. Overexpression of miR-1296 was able to significantly reduce MCM2 expression, indicating that miR-mediated “down-regulation of oncogenes might be a novel therapeutic approach [98].

Aplasia Ras homolog member I (ARHI) is a small G protein with tumor supressor activity. Low expression of ARHI is associated with shorter progression-free survival in pancreatic cancer [115]. Overexpression of ARHI, which was found to be also lowly expressed in prostate cancer cell lines and tissues, in PC-3 cells inhibited cell proliferation, colony formation and invasion ability while inducing apoptosis. ARHI is regulated by promoter CpG methylation and post-transcriptional targeting by miRNAs 221/222. Both oncogenic miRNAs are up-regulated in PC-3 cells and were shown to be overexpressed in CLL as well as thryoidand hepatocellular carcinoma [113]. GEN at a concentration of 50 μM was able to reduce miR-221/222 expression and therefore up-regulate ARHI levels, suggesting that the anti-proliferative and pro-apoptotic effect of GEN might be mediated via targeting the epigenetic regulation of ARHI [99].

Another miRNA up-regulated in prostate cancer is miR-151, which consists of the two mature miRNAs miR-151-3p and -5p. Chiyomaru *et al.* [100] demonstrated that GEN (25 μM) significantly decreased miR-151 levels in PC-3 cells. MiR-151-5p knockdown repressed cell migration, while miR-151-3p knockdown had no significant effect. Two of the targets of miR-151 are SOX17, a candidate tumor suppressor and inhibitor of Wnt-signaling in colorectal cancer and hepatocellular carcinoma, and ARHGDIA, a negative regulator of Rho GTPases. Loss of ARHGDIA is associated with enhanced metastasis and tamoxifen-resistance in breast cancer. Both target genes showed significantly reduced expression in miR-151-5p transfected prostate cancer cell lines, suggesting that GEN-mediated down-regulation of miR-151 is able to target SOX17 and ARHGDIA [100].

Linking two epigenetic mechanisms, miR-29a and miR-1256 were shown to be down-regulated in prostate cancer cell lines by promoter methylation, compared to normal prostate epithelial cells [101]. Treatment with G2535, a mixture of 70.5% GEN, 26.3% DAI and 0.31% GLY, led to promoter demethylation and increased expression of both miRNAs. TRIM68, a ubiquitin E3 ligase that acts as a co-activator of the AR and is up-regulated in prostate cancer, was confirmed as a target for miR-29a and miR-1256. Overexpressing any of the two miRNAs or G2335 treatment down-regulated TRIM68 expression and inhibited cell proliferation and invasion [113].

In two additional studies by Chiyomaru *et al.*, GEN was able to up-regulate miR-574-3p [102] and down-regulated the lncRNA HOTAIR (HOX antisense intergenic RNA) [103] in cell culture. miR-574-3p targets include the GTPase RAC1, epidermal growth factor receptor (EGFR) and the HAT p300. When these genes were knocked down by siRNA constructs, prostate cancer cells showed significantly reduced proliferation and significantly decreased invasion ability. The oncogenic lncRNA HOTAIR, in interaction with PRC2 and the lysine specific demethylase 1 (LSD1) couples H3K27methylation and H3K4 demethylation for epigenetic silencing of a multitude of genes [44,116]. HOTAIR is highly expressed in several cancer types (prostate, breast, colorectal, liver, pancreas, laryngeal cancer) and is associated with poor outcome and metastasis in breast cancer. Chiyomaru *et al.* demonstrated that GEN up-regulates miR-34a, which might then target and repress HOTAIR expression. These results showcase that GEN is able to induce tumor suppressing mechanisms by concomitant targeting of distinct mechanisms involved in cancer-associated pathways.

miR-1260b is an onco-miR up-regulated in prostate cancer. GEN (25 μM) significantly decreased espression of this onco-miR in prostate cancer cell lines. Overexpression of miR-1260b in a normal prostate cell line increased cell proliferation, whereas knock-down decreased proliferation as well as invasion ability and increased the number of apoptotic PC-3 cells. Hirata *et al.* confirmed SFRP1 (a Wnt-antagonist) and Smad4 (transforming growth factor beta signalling mediator) as targets of miR-1260b. Both genes are present at low levels in cancer tissues and were up-regulated in cancer cell lines upon GEN treatment. GEN reduced promoter methylation of SFRP1 and decreased levels of repressive H3K9me2, -me3 and H3K27me3 at the SFRP1 and Smad4 promoters, suggesting that GEN is able to activate both genes through all three epigenetic mechanisms [104].

These studies suggest that integrated analyses of all major epigenetic mechanisms are required to comprehend the impact of GEN or combinations of IF on epigenetic gene regulation, and to assess their relevance for cancer preventive potential *in vivo*.

#### 2.3.2. Kidney

Renal cancer accounted worldwide for 338000 cases and 144000 deaths in 2012 [1,11]. An estimated 63920 people in the US will develop this disease in 2014, and 13860 are expected to die from it [50]. Overall 5-year survival rates are around 50%. Among other causes, smoking and obesity are considered as major risk factors [1,2].

Similar to their studies in prostate cancer cell lines [91], Majid *et al.* analyzed epigenetic mechanisms involved in regulation of BTG3 expression targeted by GEN in three renal cancer cell lines. GEN treatment of A498, ACHN and HEK-293 cells at 10–50 μM dose-dependently decreased cell growth, cell cycle progression and promoter methylation of BTG3, leading to a pronounced mRNA re-expression by 50 μM GEN. Using several activity kits for epigenetic enzymes, they found that DNMT activity was reduced, with a significant decrease in DNMT3b protein expression. HDAC activity and MBD2 binding to the BTG3 promoter were also reduced, whereas HAT activity was increased. Immunoprecipitation analysis discovered an overall increase in acH3, acH4, H3K4me2 and H3K4me3, representing active histone mark assembly at the promoter region of BTG3. H3K9me2 and H3K9me3 as repressive marks were decreased, but solely in ACHN cells [105].

Oncogenic miR-21 is overexpressed in many human cancer types (glioblastoma, lung, colon, pancreas, liver, esophagus, skin, stomach and prostate cancers, as well as some AML cases). Its targets include PTEN, EGFR, and CDK6, among others, suggesting its important role in cancer development [82,113]. A study by Zaman *et al.* correlated kidney cancer survival rates with miR-21 expression levels. In this study, all patients with low miR-21 levels, but only 50% of the patients with high miR‑21 levels survived five years after surgery [106]. Knockdown of miR-21 in renal cancer cell lines caused repressed cell growth, invasion and migration as well as up-regulation of the cell cycle inhibitor p21 and down-regulation of several cyclin-dependent kinases such as CDK2, CDK4, CDKE1 and CDKE2, suggesting that miR-21 directly or indirectly regulates these genes. Pretreatment of A-498 cells with 25 μM GEN for four days prior to subcutaneous injection into nude mice significantly reduced xenograft size and miR-21 levels, suggesting that GEN might inhibit renal tumor development and growth through down-regulation of miR-21 [106].

In a second study in 2012, the same authors reported that also miR-23b-3p levels were correlated with survival in patients with renal carcinomas. As with miR-21, all patients with low or moderate miR-23b-3p expression, but only half of the patients with high miR-23b-3p expression survived five years post-surgery. miR-23b-3p knockdown in renal cancer cells resulted in an increased number of apoptotic cells and decreased cell invasion and migration ability. Tumor suppressor PTEN was increased in miR-23b-3p knockdown cells and absent in tumor samples with high miR-23b-3p expression. The 3’UTR of PTEN was shown to be a direct target for miR-23b-3p, suggesting PTEN is the mechanism through which miR-23b-3p induces apoptosis. GEN treatment decreased miR-23b-3p expression, indicating that the pro-apoptotic effect of GEN on renal cancer cells could be caused by down-regulated miR-23b-3p expression [107].

MiR-1260b is significantly higher expressed in renal cancer tissues compared to normal kidney and even higher in stage 2, 3 and 4 patients compared to stage 1 patients. High miR-1260b levels also correlated with shorter survival. Transfection of renal cancer cell lines with pre-miR-1260b increased cell viability and invasion and repressed apoptosis. GEN down-regulated miR-1260b expression as well as Wnt-signaling [108]. This might be due to the fact that miR-1260b targets SFRP1, a Wnt-antagonist, as described previously [104].

Similar to reports in prostate cancer, the lncRNA HOTAIR is significantly higher expressed in renal carcinoma cells compared to normal kidney, whereas miR-141 is significantly lower expressed, suggesting a link between the two RNAs. Indeed, miR-141 was shown to bind to and suppress HOTAIR expression. Treatment with GEN induced miR-141, and consequently repressed HOTAIR expression. In pre-miR-141 transfected cells, ABL2 (c-Abl oncogene 2, a non-receptor tyrosine protein kinase) and proto-cadherin 10 (PCDH10) expression was reduced, while Snail expression was induced, suggesting GEN targets EMT regulating mechanisms in renal cancer [109].

The group of Rajvir Dahiya from the University of California in San Francisco (USA) has made numerous contributions to elucidating miR-related and other mechanisms of epigenetic gene regulation by GEN, especially in urological cancers. Overall, these studies indicate that GEN and other IF affect a multitude of miRNAs and the lncRNA HOTAIR in prostate and kidney cancer cells, although in most studies, the used concentrations were relatively high. One important area of research that is still unresolved is how GEN and other dietary agents modulate the expression of selected miRs. Some studies have indicated that demethylation of silenced miRNA promoters contributes to the observed expression of tumor suppressor miRs. However, future studies will have to elucidate how dietary agents such as IF can reduce expression of onco-miRs, and whether the described regulation of ncRNAs is relevant *in vivo*.

### 2.4. Gastrointestinal Tract

While cancer prevention of most solid tumors requires systemic uptake of dietary factors for their delivery to target organs, the gastrointestinal tract (GIT) is an exception since it comes into direct contact with plant secondary metabolites, such as IF and other dietary compounds during their passage through the body after ingestion. GIT cancers include cancers of the esophagus, stomach, pancreas, liver, gallbladder, colon and rectum. Studies on IF-mediated effects in the GIT are summarized in Table 4.

#### 2.4.1. Esophagus

With 456000 cases and 401000 deaths in 2012, esophageal cancer accounted for 3.2% of all cancers worldwide [1,11]. Major risk factors include tobacco and alcohol consumption [2]. Esophageal cancer is usually lethal with only a 5% survival rate over five years, and is the leading cause of cancer-related death in China [2].

In an early study by Fang *et al.*, two human esophageal squamous cell carcinoma cell lines KYSE 150 and 510 were subjected to dose- and time-dependent GEN treatments [81]. Investigating RARβ2, the cell cycle regulator p16 (also known as CDKN2a, Cyclin-dependent kinase inhibitor 2A) and the DNA repair gene MGMT (O_6_-methylguanine-methyltransferase), Fang *et al.* observed reduced promoter methylation and mRNA re-expression in a dose- and time-dependent manner. Treating cells with TSA and decitabine or both in combination with GEN significantly enhanced reactivation of the tumor suppressor genes, suggesting a synergistic mode of action. HDAC and DNMT activity assays revealed dose-dependent inhibitory features of GEN [92].

**Table 4:** Soy isoflavones targeting epigenetic mechanisms in the gastrointestinal tract (GIT) *in vitro* and *in vivo*.

Organ	Compounds and Concentration/Dose Tested	Incubation Time	Cell Lines—*In Vivo* Models	Genes Regulated and Underlying Mechanisms	Methods Used—Comments	First Author, Year [Reference]
**Esophagus**	GEN 2, 5, 10, 20 μM DAI 5, 10, 20 μM	1, 2, 4, 6 day	KYSE 150, KYSE 510	↓ RARβ2, p16, MGMT promoter methylation ↑ RARβ2, p19, MGMT expression ↓ DNMT and HDAC activity (weak) ↓ cell growth ↔ DNMT and MBD2 mRNA expression	MSP RT-PCR, RT-qPCR DNMT activity assay with [^3^H]-SAM HDAC Kit (Upstate)	Fang, 2005 [92]
**Stomach**	GEN 10, 25, 50 μM	3 days	AGS	↓ PCDH17 promoter methylation ↑ PCDH17 expression	BS, RT-qPCR	Yang, 2012 [117]
**Colon**	GEN 25 μM	3 days	HCT 116, HT-29	↓ RARβ2 promoter methylation	BS	Spurling, 2008 [118]
	Novasoy Extract 200 μg/mL, GEN 75 μM	4 days	SW1116, SW480, DLD-1	↓ WNT5a promoter methylation, ↑ WNT5a expression (in SW1116 GEN) ↓ cell viability	MSP, Methylation-sensitive restriction enzyme PCR (MSREP), BS	Wang, 2010 [119]
	GEN 75 μM	4 days	DLD-1	↓ SFRP2 promoter methylation ↑ SFRP2 expression ↓ cell proliferation ↑ apoptosis	MSP, RT-qPCR	Zhang, 2011 [120]
	GEN 50, 75 μM	2 days, 4 days	SW480, DLD-1, HCT-15, HT-29, RKO, SW48	↔ DKK1 promoter methylation, ↑ DKK1 expression (SW480) ↓ proliferation, ↑ cell cycle arrest in G2/M phase (SW480) ↓ cyclinD1 mRNA, ↔ p21, cMyc mRNA ↑ acH3 at DKK1 promoter ↔ acH4, H3K4me2	MSP, BS RT-qPCR DKK1 knockdown and overexpression, ChIP-PCR	Wang, 2012 [121]
	SPI (140 mg/kg GEN) Casein protein plus 140 mg/kg GEN; AOM challenge	Prenatal until PNW 14	Sprague-Dawley rats	↓ AOM-induced promoter demethylation ↓ AOM-induced expression of Sfrp2, Sfrp5 and Wnt5a ↓ RNA PolII binding to Sfrp2 (GEN), Sfrp5 and Wnt5a promoter ↓ AcH3, H3K9me3, H3S10P at Sfrp2, Sfrp5, Wnt5a promoter ↑ nuclear HDAC3 expression	MSP, BS RT-qPCR ChIP-PCR Western blotting	Zhang, 2013 [122]
	GEN 10–200 μM	1 day	HT-29	↓ HDAC1 expression and activity (IC_50_ 97 μM) ↔ no cytotoxicity in the presence of catalase to remove artifactual hydrogen peroxide	HDAC assay kit (Cayman) Sulforhodamin B staining WST-1 (water soluble tetrazolium) assay Western blotting	Groh, 2013 [123]
**Pancreas**	G2535 (equivalent to 10 μM GEN)	3 weeks	AsPC-1, MiaPaCa-2, Panc-1 L3.6pl, Colo357, BxPC-3, HPAC	altered expression of 67/711 miRs ↑ miR-200 and let-7 family members ↓ EMT phenotype and markers ↑ sensitivity to gemcitabine in gem. resistant cells	miRNA microarray RT-qPCR Western blotting	Li, 2009 [124]
	G2535 (equivalent to 25 μM GEN)	2 days	Colo357, Panc-1, HPDE	↑ miR-146a expression ↓ EGFR, IRAK-1, NF-κB and MTA-2 expression ↓ cell invasion	miRNA microarray RT-qPCR Western blotting	Li, 2010 [125]
	GEN 60 μM	16 h, 3 days, 7 days	AsPC-1, MiaPaCa-2	↑ miR-34a expression ↓ Notch-1 expression ↓ cell growth ↑ apoptosis	RT-qPCR Western blotting	Xia, 2012 [126]
	GEN 60 μM	3 days	AsPC-1, BxPC-3	↓ miR-223 expression ↑ Fbw7 protein expression ↓ cell growth ↑ apoptosis ↓ migration and invasion	MTT assay FACS for apoptosis detection RT-qPCR Western blotting	Ma, 2013 [127]
	GEN	n.a.	n.a.	↓ miR-27a ↓ cell growth ↑ apoptosis ↓ invasion	MTT assay RT-qPCR Western blotting	Xia, 2014 [128] (abstract only)
	Equol 10, 100 μg	PND 1–10	Sprague-Dawley rats	↑ methylation at the c-Ha-Ras gene	methylation sensitive restriction digestion, Southern blotting	Lyn-Cook, 1995 [80]

Abbreviations: SPI: soy protein isolate; AOM: Azoxymethane; G2535: contains 70.5% GEN, 26.3% DAI, 0.3% GLY); HPDE: Human pancreatic duct epithelial cells; also see footnotes Table 1.

#### 2.4.2. Stomach

In 2012, stomach cancer had a global incidence of 952000 cases and resulted in 724000 cancer deaths [1,11]. This cancer type has mainly been associated with bacterial infections with *Helicobacter pylori*. Epidemiological studies suggest that consumption of soy and soy products decreases stomach cancer risk [2].

Yang *et al.* investigated the effects of GEN in the gastric cancer cell line AGS. PCDH17 (Protocadherin 17), a tumor suppressor frequently methylated in gastric and colorectal cancers, was highly methylated in AGS cells. Treating the cells with GEN or decitabine induced hypomethylation at several CpG sites, resulting in re-expression of PCDH17 mRNA with more pronounced effects in GEN-treated cells [117].

#### 2.4.3. Colon

Colorectal cancer is the third most common cancer type in males, and the second most common cancer type in females, with a global incidence of 1,360,000 cases and 694,000 deaths in 2012 [1,11]. In the US, colorectal cancer is the fourth most common cancer type. In 2014, it is estimated that there will be 136,830 new cases, and an estimated 50,310 people will die of this disease [50]. In comparison to other tumors of the GIT, colorectal cancer has a relatively high 5-year survival rate of about 50%. Lifestyle factors including diet, obesity, smoking and lack of physical activity, as well as increasing age have been identified as major risk factors, making colorectal cancer a generally preventable disease. Highest incidence of colorectal cancers was reported in high-income countries, but this cancer type is on the rise in mid- and low-income areas while being rather uncommon in Asia and Africa [1,2].

Similar to breast, prostate and cervical cancer, RARβ2 is commonly silenced in colon cancer through promoter hypermethylation. Spurling *et al.* investigated demethylating properties of all-trans retinoic acid (ATRA) and butyrate in combination with GEN in HCT 116 and HT-29 cells. A combination of all three agents massively induced RARβ2 mRNA expression, but treatment with GEN alone most efficiently reduced hypermethylation at the promoter region [118].

Genetic or epigenetic alterations in the Wnt-signaling pathway play a pivotal role in the development of colon cancer. Wnt-proteins are a family of secreted signaling molecules that bind to receptors of the Frizzled and low-density lipoprotein receptor-related protein families on the cell surface and regulate cell-to-cell interactions during embryogenesis. Signals of the Wnt-signaling pathway are transmitted through several cytoplasmic components to β-catenin. In the presence of a Wnt-ligand, β-catenin accumulates and enters the nucleus, serving as a co-activator for TCF (T-cell factor) transcription factors for active transcription of Wnt‑responsive genes. Signaling through the Wnt-pathway is a fundamental mechanism to control cell proliferation and development, and as a result deregulation is often linked to cancer [129]. WNT5a, a Wnt-ligand of the non-canonical Wnt-pathway, can both promote and repress Wnt-signaling. Increased expression of WNT5a has been shown to promote EMT and metastasis in pancreatic cancer cells [130], while inhibiting EMT, cell proliferation and invasion in colon cancer [131]. In various colon cancer cell lines, WNT5a expression was inversely correlated with promoter methylation. Treatment with GEN or a commercial soy extract (Novasoy) led to demethylation and re-expression of the protein, affecting especially those cell lines with hypermethylated WNT5a promoter, whereas no changes in expression were observed in cell lines with unmethylated WNT5a promoter [119]. In line with these data, Zhang *et al.* reported that SFRP2, a member of the secreted frizzled related family of Wnt-antagonists, was re-expressed in the DLD-1 colon cancer cell line by 75 μM GEN through promoter demethylation (measured by MSP) [120]. GEN treatment led to reduction of cell proliferation and induction of apoptosis.

DKK1 (Dickkopf-related protein 1) is another inhibitor of Wnt-signaling. Silencing of DKK-1 in colon cancer has been associated with microsatellite instability; its expression is regulated by both promoter methylation as well as histone tail modifications [121]. GEN treatment led to re-expression of DKK1 in SW480 and HCT-15 cell lines, whereas mRNA levels were not affected in other colon cancer cell lines, especially those with extensive DKK1 promoter methylation (RKO, SW48, DLD-1). Independent of the methylation status, GEN did not affect DKK1 promoter methylation in any tested cell line. In SW480 cells, GEN dose- and time-dependently induced DKK1 mRNA and protein levels. Overexpression and knockdown of DKK1 mimicked and reversed, respectively, GEN-mediated cell-cycle arrest in G_2_/M phase, cell proliferation, and reduction of cyclinD1 mRNA levels. Mechanistically, GEN induced acetylation at histone H3 (H3ac), but not at histone H4 in SW480 cells, with no significant changes in histone acetylation in DLD-1 colon cancer cells. Histone methylation (H3K4me2) and phosphorylation of H3 at serine 10 (H3S10P) were not affected by GEN. Similar to GEN-mediated induction of H3 acetylation, inhibition of deacetylase activity by TSA efficiently induced DKK1 transcription [121].

After having identified the influence of GEN on epigenetic regulation of Wnt-antagonists *in vitro*, Chen and co-workers were also interested in its potential to affect epigenetic mechanisms during colon carcinogenesis *in vivo* [122]. Spraque Dawley rats life-long exposed to a GEN- or soy protein isolate (SPI)-containing diet *vs*. a casein protein-containing control (CTL) diet were challenged twice with the colon carcinogen azoxymethane (AOM) and sacrificed six weeks later, modeling initial stages of colon carcinogenesis. Earlier studies had shown that these interventions reduced AOM-induced pre-neoplastic aberrant crypt foci (ACF) and nuclear accumulation of β-catenin in the rat colon [132]. Pre-AOM samples were obtained from rats at the age of seven weeks. Lack of methylation at specific regions in the promoters of the Wnt-antagonists SFRP2 and 5 as well as WNT5a (determined by MSP with primers for unmethylated DNA) correlated with gene expression. AOM treatment decreased methylation at the SFRP5 (secreted frizzled related protein 5) promoter; this was prevented by both GEN- and SPI interventions. GEN also prevented the AOM-induced demethylation at the SFRP2 promoter compared to CTL and the GEN-exposed pre-AOM group. Overall, intervention with both diets reduced polymerase II (PolII) binding to the promoters of all three genes, indicating reduced transcriptional activity. AOM significantly increased levels of H3ac and the two neighboring and interacting modifications H3K9me3 (repressive mark) and H3S10 phosphorylation (mitotic mark) compared to CTL at all three genes. Both diets reduced these marks post-AOM. GEN-diet alone increased H3S10 phosphorylation in pre-AOM animals at all three genes, indicating mitotic arrest, and also significantly reduced H3ac at the SFRP2 promoter, whereas DNA methylation in this region slightly increased. Changes in H3ac were explained by an increase in nuclear levels of HDAC3 protein in post-AOM samples of animals exposed to GEN- or SPI-diets.

Overall, these data indicate that Wnt-signaling is activated by AOM treatment in early colon carcinogenesis. Epigenetic remodeling by life-long GEN or SPI exposure represses Wnt-signaling and activation of β-catenin; however, these effects are not due to an up-regulation of the Wnt antagonists SFRP2, SFRP5 and WNT5a. Rather, GEN or SPI maintain Wnt-signaling genes at levels detected in normal colon. These effects are different from those observed in colon cancer cell lines, where GEN treatment suppressed aberrantly activated Wnt-signaling by up-regulating Wnt antagonists [119,120,121]. Chen *et al.* speculate that complex interactions of histone tail modifications and DNA methylation are involved in the control of Wnt-related gene expression by GEN and SPI.

#### 2.4.4. Pancreas

In 2012, pancreatic cancer accounted worldwide for 338,000 cases and 331,000 deaths; it mainly occurs in high-income countries. Since it is generally diagnosed very late in the progression of the disease, pancreatic cancer is almost always lethal with a 5-year survival rate of only 4.1% [1,2,11]. The aggressiveness of pancreatic cancer is partly due to acquisition of drug resistance characteristics, which are also associated with epithelial-mesenchymal transition (EMT) [125].

EMT is thought to be an important mechanism involved in cancer metastasis [133,134,135]. Both loss of cell adhesion as well as down-regulation of epithelial markers, such as *E*-cadherin, and up-regulation of mesenchymal markers, including *N*-cadherin, vimentin and fibronectin are characteristics of cells undergoing EMT. Although EMT is an essential process for development, cancer cells can abuse this process to increase invasiveness and motility, ultimately leading to metastasis [133]. MiRNAs, especially the miR-200 family [136,137], were shown to regulate EMT associated TFs, such as Snail, Slug, Twist, ZEB1 (Zinc Finger E-Box Binding Homeobox 1, also known as TCF8 or Delta EF1) and ZEB2. Once cancer cells have undergone EMT they need to go back to an epithelial-like state in order to form a secondary tumor. This process is called mesenchymal-epithelial transition (MET) [135]. The fact that miRNAs can regulate EMT-promoting factors might indicate that they also can regulate MET-promoting factors, underlining their potential importance as a targets for cancer treatment and prevention (reviewed in [138]).

In 2009, Li *et al.* reported that gemcitabine-resistant pancreatic cancer cells had a different miRNA expression profile than gemcitabine-sensitive cell lines. Most strikingly, the known EMT-regulating miRNAs of the miR-200 family as well as many members of the tumor suppressing let-7 family were down-regulated in the resistant cell line. Indeed, transfecting gemcitabine-resistant cells with miR-200a, -200b and -200c as well as G2535-treatment (IF mix of 70.5% GEN, 26.3% DAI, 0.3% GLY, dose equivalent to 10 μM GEN) caused repression of the EMT phenotype and mesenchymal markers (such as vimentin, ZEB1, Slug, Twist) and increased the expression of epithelial markers (such as E-cadherin). Furthermore, G2535 increased sensitivity to gemcitabine in gemcitabine-resistant cancer cells. These findings indicate that G2535 could have an anti-EMT effect and be beneficial for a gemcitabine therapy [124].

Another study by Li *et al.* [125] demonstrated that G2535 treatment (equivalent to 25 μM GEN) of pancreatic cancer cells up-regulated the expression of miR-146a, involved e.g., in innate immunity. Transfection with pre-miR-146a inhibited expression of EGFR, IRAK-1 (Interleukin-1 receptor-associated kinase 1) involved in NF-κB signalling, and the pro-inflammatory TF NF-κB and MTA-2 (Metastasis-associated protein 2), a predicted target of miR-146a and reduced pancreatic cell invasion capacity. Similarly, G2535 inhibited EGFR, IRAK-1/NF-κB and MTA-2 expression, suggesting that up-regulation of miR-146a by G2535 might be mechanistically involved in inhibition of invasion [125].

Several reports indicate that p53 directly regulates the miR-34 family, including miR-34a, and that some effects of p53 could be caused by these miRNAs [113]. In 2012, Xia *et al.* demonstrated that GEN (60 μM) inhibited cell growth, clonogenity, migration and invasion, while inducing apoptosis in pancreatic cancer cell lines. They also found that miR-34a is down-regulated in these cancer cell lines and was significantly up-regulated upon GEN treatment. Expression of Notch-1, a target of miR-34a was decreased in GEN-treated and in miR-34a-transfected cells, suggesting that the effects of GEN may be caused by miR-34a up-regulation and Notch-1 repression [126].

miR-223 is up-regulated in various tumor types and plays an oncogenic role in pancreatic cancer. One of its target genes is Fbw7, a ubiquitin E3 ligase that targets several oncogenes including Notch-1, Mcl-1, c-Myc and Cyclin E and marks them for proteasomal degradation. Ma *et al.* showed that GEN (60 μM) down-regulated miR-223 expression, reduced cell growth and invasion, and increased apoptosis in pancreatic cancer cell lines. GEN in combination with anti-miR-223 was even more effective in inducing Fbw7 levels, and consequently inhibiting cell growth, migration as well as invasion and inducing apoptosis, indicating that some of the effects of GEN might be through miR-223 repression and induction of its target Fbw7 [127].

A very recent publication by Xia *et al.* (abstract only) indicated that similar effects of GEN on pancreatic cancer cells, including decreased proliferation, migration, invasion and increased apoptosis were partly caused by down-regulation of miR-27a [128]. As reported above, the influence of GEN on miR-27a was previously investigated by this group in ovarian cancer cells [78] and uveal melanoma [79].

As these studies indicate, GEN and the IF extract G2535 seem to possess promising activity in inhibiting proliferation, migration, and invasion and inducing apoptosis in pancreatic cancer cell lines through modulation of the expression of miRNAs. Also, EMT and the development of metastasis could be important miR-regulated mechanisms targeted by GEN in panceatic cancer. In some studies, the concentrations used were still relatively high, and we have to await further confirmation of the reported effects in animal studies. However, due to the high mortality rate of pancreatic cancer, nontoxic intervention with IF to prevent or reduce drug resistance and meatastases might be a strategy worth of consideration for the prevention or adjuvant therapy of pancreatic cancer.

Apart from miRNA-mediated effects, already in 1995, Lyn-Cook *et al.* were interested in DNA methylation changes of various proto-oncogenes in pancreatic tissue of Sprague-Dawley rats after neonatal intervention on PND 1–10 with equol, a metabolite of DAI produced by the gut microbiota. Using methylation-sensitive restriction digestion and Southern blotting, they demonstrated that equol at two doses (10 and 100 μg) increased methylation at the c-Ha-Ras gene (Harvey rat sarcoma viral oncogene homolog) in tissues of rats sacrificed at PND 15, whereas no changes were observed at the c-Myc or c-Fos proto-oncogenes [80].

### 2.5. Further in Vivo Studies on Epigenetic Effects of IF

A summary of these studies is given in Table 5.

**Table 5:** Soy isoflavones targeting epigenetic mechanisms in further *in vivo* studies.

Organ	Compounds and Concentration/Dose Tested	Incubatio*n* Time	Cell lines—*In Vivo* Models	Genes Regulated, Underlying Mechanisms	Methods Used—Comments	First AutorYear [Reference]
**Leukemia**	GEN 0.1, 1, 10 μM *In vivo:* GEN 0.5% (w/w) in the diet	2 days, whole lifespan	HL-60, L1210, L1210/ARA-C; CD2F1 male mice	↑ loss of clonogenicity ↓ p57 promoter methylation ↔ 5-Aza-CdR incorporation ↑ survival time (mice)	Colony forming Assay, [6-^3^H]5-Aza-CdR incorporation, MSP, UPLC	Raynal, 2008 [139]
**Neuro-blastoma**	GEN *In vivo*: 2 mg/mouse/day	15 days	SK-N-SH Neuroblastoma xenografts	↓ xenograft tumor size and frequency ↓ CHD5 promoter methylation ↑ CHD5 mRNA expression ↓ DNTM3b mRNA expression	BS, RT-qPCR, Western blotting	Li, 2012 [140]
**Agouti mice**	GEN *In vivo:* 250 mg/kg diet	prenatal until PND 21	A^vy^mice (tail clips, liver, brain, kidney	↑ methylation of CpG sites in the cryptic promoter region of A^vy^ IAP shifting coat color to pseudo agouti (less obese) ↔ coat color ↔ coat color	BS	Dolinoy, 2006 [141] Badger, 2008 [142] Rosenfeld, 2013 [143]
**ESC**	GEN 5 μM	4 days	CGE, E14Tg2a	149 differentially methylated regions: 54 hyper- and 95 hypomethylated ↑ Ucp1 and Sytl1 demethylation during ESC differentiation after initial *de novo* methylation	MspI fragment based DNA methylation typing (MFMT), BS	Sato, 2011 [144]
**Blood, Bone marrow**	GEN *In vivo:* 270 mg/kg diet	prenatal until PND 0	123/SvJ:C57BL/6J mice	↑ methylation in repetitive elements in adult mice (not in fetal mice) ↓ HDAC6, p21, cyclin D1, PCNA, and IGF2 expression in adult mice ↑ switch from primitive erythroid to definite erythroid lineage (blood cell development)	Methylation-sensitive McrBC real time PCR assay, Agilent Microarray 4x44k	Vanhees, 2011 [145]
**Water flea**	GEN In culture media 4.7 mg/L (3/week for 3 weeks)	21 days, multi-generational	*Daphnia magna*	↓ reproduction in F_0_ ↓ body length in F_0_, F_1_ and F_2_ ↔ global DNA methylation	UPLC	Vandegehuchte, 2010 [146]
**Adipose tissue, muscle, (liver, blood)**	TAD TAD + IF equivalent to 180 mg/person/day	8 weeks, plus change of chow for 8 weeks	Cynomolgus monkeys	Methylation of HOXA5, HOXB1, HOXA11, NTRK3, PLAG12A, ABCG5, TBX5 differed significantly in muscle or fat tissue ↓ fasting insulin levels ↑ improved insulin sensitivity	Illumina 27k array PyroSequencing	Howard, 2011 [147]
**PBMCs**	soy rich diet (~230 mg IF/day)	4 weeks	Heavy smokers	↑ Line-1 methylation ↓ inter-individual methylation variability ↔ MTHFR, MLH1, RASSF1A, CDN2A, ARF	PyroSequencing	Scoccianti, 2011 [148]

Abbreviations: ESC: embryonic stem cells; TAD: typical (high fat) American diet; PBMC: peripheral blood mononuclear cells; ULPC: Ultra Performance Liquid Chromatography; also see footnote Table 1

#### 2.5.1. Anti-Cancer Treatment of Leukemia and Neuroblastoma in Mouse Models

Decitabine is approved in the clinics for the treatment of myelodysplastic syndromes and acute myeloid leukemia. Raynal *et al.* performed a study with human and murine leukemic cell lines and CD2F1 mice infused with leukemic cells to investigate whether co-treatment with decitabine and GEN might result in synergistic effects. A synergistic loss of clonogenicity of both decitabine-sensitive and -resistant leukemic cells indicated that GEN might significantly enhance the anti-leukemic properties of decitabine. Co-treatment also re-activated mRNA expression of the tumor supresssor gene p57, accompanied by a dose-dependent reduction of p57 methylated band intensity determined by MSP. Co-treatment also increased survival of mice after infusion of leukemic cells as compared to decitabine alone. Since decitabine incorporation into DNA was not influenced by GEN treatment, the mechanism underlying the improved effects after co-treatment still needs to be elucidated [139].

Neuroblastoma is a neuroendocrine tumor that mainly develops in children. In a xenograft model with neuroblastoma cells, GEN treatment (2 mg/mouse/day) for 15 days reduced the average tumor size and microvessel density (a marker for angiogenesis) and increased p53 expression. CHD5 (chromodomain-helicase-DNA-binding protein 5) is a downstream actor in the p53 pathway and a potential TSG that is deleted or epigenetically silenced in neuroblastoma. GEN treatment led to down-regulation of DNMT3b and reduced CHD5 promoter methylation (determined by BS). As a consequence, CHD5 mRNA and protein expression were up-regulated in neuroblastoma tumors and might contribute to inhibition of cell proliferation [140].

#### 2.5.2. Developmental/Multi-Generational Effects in Various Models

The Agouti mouse is a rodent model that allows phenotypical detection of alterations in DNA methylation induced by environmental exposure to epigenetically active agents [149]. The murine Agouti gene encodes for a paracrine signal that promotes yellow over black coat color. The expression is epigenetically regulated by a retrotransposon upstream of its transcription start site and correlates well with ectopic transcription leading to yellow fur, obesity and tumorigenesis. Dolinoy *et al.* investigated the effect of prenatal maternal exposure of GEN in this model. Prenatal GEN exposure (250 mg/kg diet) shifted coat color from yellow agouti towards pseudo agouti, indicating a reduced expression of the Agouti gene. GEN exposure also increased methylation of six CpGs in the retrotransposon of the offspring [141]. However two recent studies using the same model, published by Badger *et al.* [142] and Rosenfeld *et al.* [143], did not confirm significant alterations in coat color after GEN exposure of Agouti mice.

Based on the studies with GEN in Agouti mice, Sato *et al.* were interested in identifying the developmental time window during embryonic development that might be most susceptible to epigenetic changes by GEN exposure. They employed a mouse embryonic stem cell (ESC) differentiation system mimicking the post-implantation period with active *de novo* DNA methylation. Cells were treated *in vitro* with 5 μM GEN for four days, and “MspI fragment-based DNA methylation typing” (MFMT) was utilized for genome-wide methylation analyses. They identified 149 differentially methylated regions (54 hyper- and 95 hypomethylated) in comparison with a solvent control. Two genes, the mitochondrial uncoupling protein Ucp1 and Sytl1 (Synaptotagmin-like 1), thought to be involved in vesicular trafficking, were selected for detailed methylation analyses by BS as representatives of genes with low (Sytl1) and intermediate (Ucp1) CpG dense promoters. GEN treatment did not affect the peak of promoter methylation occurring at day 4, but accelerated the decline of DNA methylation until day 10 of differentiation. The authors concluded that GEN is able to perturb methylation patterns after initial *de novo* methylation during ESC differentiation [144].

Prenatal deficiencies, for example of folate and vitamin B_12_, or exposure to estrogenic compounds can affect blood cell composition. Therefore it was of interest whether prenatal exposure to GEN might alter hematopoiesis. In a study of Vanhees *et al.*, maternal treatment with GEN (270 mg/kg diet) from three days before conception until the end of pregnancy led to a significant increase in granulopoiesis, erythropoiesis, and mild macrocytosis in the offspring at the adult age of 12 weeks [145]. Prenatal GEN exposure led to long-term epigenetic changes. Hypermethylation at various repetitive elements was detected in bone marrow of adult offspring, but not in fetal mice, and coincided with down-regulation of several estrogen-responsive genes, including HDAC6, p21, cyclin D1, PCNA, and IGF2, and modulation of genes involved in hematopoiesis (for example up-regulation of CDK inhibitor p27 and the TFs C/EBPα and -β). The results emphasize the ability of GEN to leave a permanent signature on the epigenome in a maternal IF exposure scenario, accompanied by a switch in blood cell development from primitive erythroid towards definite erythroid lineage [145].

Water fleas (*Daphnia magna*) are considered to be a good model system for environmental risk assessment. Vandegeruchte *et al.* compared in a multi-generation experiment the effects of the methylation inhibitor 5-azacytidine and GEN on reproductive features and changes in global DNA methylation [146]. Animals were exposed to GEN (4.7 mg/L culture media) either for one generation leaving two daughter generations unexposed or for a total of three generations. The F_0_ generation showed a reduced reproduction rate and body length, which was maintained in continuously GEN-exposed but not in unexposed F_1_ and F_2_ generations. Global DNA methylation determined by measuring the percentage of 5-methyl-2’-deoxy-cytidine (mdC) was not affected. However, 5-azacytidine strongly affected reproduction and, in contrast to GEN, reduced mdC levels in exposed F_0_. This was maintained even in unexposed subsequent generations. These data indicate that the model is suitable to detect heritable epigenetic changes, but that there was no trans-generational epigenetic effect of GEN in *Daphnia* [146].

#### 2.5.3. Short-Term Intervention in Adult Non-Human Primates and Humans

Adapting lifelong to a soy-rich diet might be difficult to achieve in Western countries. Regular dietary soy uptake might be more feasible if short-term modulation of the diet would be sufficient to induce health-promoting effects. Howard *et al.* investigated such a diet-switch scenario in non-human primates [147]. Two groups of four Cynomolgus macaques each on either a soy-containing animal chow or a chow high in fructose and casein were assigned in a cross-over design for eight weeks to two high-fat diets representative of a typical American diet (TAD), with either casein or soy (equivalent to 180 mg soy IF for a human adult per day) as the protein source. Genome-wide methylation changes in blood, liver, muscle and subcutaneous adipose tissue were analyzed using HumanMethylation 27 k arrays (Illumina). A general increase in DNA methylation was observed in liver and muscle tissue when the monkeys were switched from a soy-based to a casein-based diet. Methylation levels were unchanged in blood, and slightly decreased in fat tissue. After correction for multiple testing (*p* < 0.2), few methylation changes remained statistically significant. Specifically in muscle tissue, two homeobox genes HOXA5 and HOXA11 were demethylated, while promoter methylation of the NTRK3 gene (neurotrophic tyrosine kinase, receptor, type 3) increased. In fat tissue, a switch from casein- to soy-based diet reduced methylation levels at the ABC-transporter G5 (ABCG5) and increased methylation at the promoters of HOXB1 and T-box protein 5 (TBX5), a TF regulating developmental processes. Methylation changes of these genes were confirmed by pyrosequencing. Apart from the epigenetic effects, the diet switch form casein- to soy-based diet reduced fasting insulin levels and improved insulin sensitivity. The authors concluded that additional studies should investigate long-term beneficial or potentially pathologic health consequences of soy and other dietary supplements on epigenetic regulation and human health [147].

Scoccianti *et al.* were interested in whether diets enriched with food sources of flavonoids and isothiocyanates might influence DNA methylation changes associated with smoking. They performed a 4-week dietary intervention study in a population of 88 healthy blood-donors who were all heavy smokers. The volunteers were randomly assigned to a standard diet, a diet enriched in flavonoid- and isothiocyanate-rich foodstuff such as cruciferous vegetables (61 mg flavonoids per day), or a diet enriched with (iso-)flavonoids from green tea and soy products (230 mg flavonoids per day). DNA methylation analysis was performed in peripheral blood mononuclear cells (PBMCs) collected before and after intervention for the repetitive sequence LINE1 (long interspersed nuclear element) and a series of tumor suppressor genes (RASSF1A and the cell cycle regulators p16 (CDKN2A) and ARF (alternate reading frame of the INK4a/ARF locus)), and genes involved in DNA repair (MLH1) and folate metabolism (MTHFR, methylene-tetrahydrofolate reductase). None of the selected genes showed significant methylation changes after the diet intervention. A small but significant increase of 0.8% and 2.1% in LINE1 methylation could be seen in both diet groups. Overall, a reduction of inter-individual variability in LINE1 methylation was the most pronounced effect of the diet changes [148]. It should be mentioned that the suitability of PBMCs as surrogate tissue to identify physiological relevant changes in DNA methylation is currently under debate [150,151]. Algorithms have been developed to correct for differences in white blood cell composition which might confound the results of methylation analyses [152,153,154].

## 3. Summary and Conclusions

**Figure 3:**
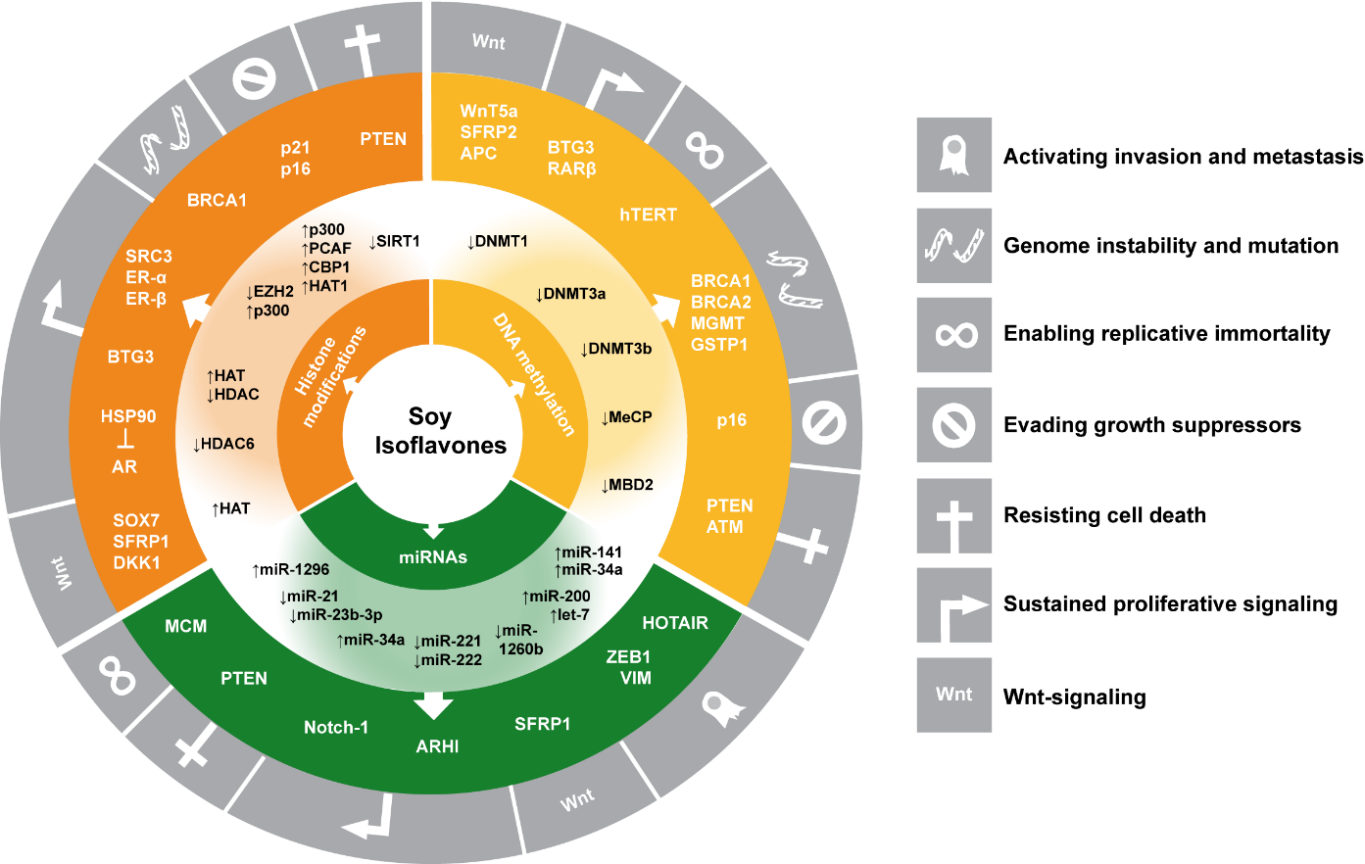
Soy IF target several “hallmarks of cancer” through epigenetic mechanisms.

During the past decade, more than 60 studies have been published that describe the potential of soy IF to affect the three key epigenetic mechanisms, DNA methylation, histone tail modifications and non-coding RNAs, and subsequently modulate gene expression to counteract the “hallmarks of cancer” [155,156,157] (overview in Figure 3).

Earlier studies focused on *DNA methylation* changes and mainly tested whether IF might be able to reverse cancer-associated aberrations in DNA methylation, especially silencing of TSGs such as RARβ2, BTG3, PTEN and ATM, genes involved in DNA repair (MGMT, BRCA1, BRCA2, GSTP1), cell signaling (especially Wnt-signaling in colon cancer), and cell cycle regulators (p16). These studies revealed demethylating activity of GEN and other soy IF, but some of the data were not consistent between different cell lines or could not be confirmed. One of the reasons might be that in early studies methodology used to demonstrate changes in DNA methylation (e.g. MSP) did not allow a true quantitative assessment of DNA methylation levels, and small changes in DNA methylation might have been over-interpreted. More recent studies used quantitative methods such as pyrosequencing and BS and therefore might be more reliable. When elucidating mechanisms underlying demethylating activity, IF were shown to lower the activity of DNMTs in cell culture. Since reduced levels of enzymatic activity can result from direct enzyme inhibitory effects, but also (and maybe more often) from changes in gene or protein expression, expression levels should be analyzed in parallel.

With few exceptions, studies mainly focused on selected candidate genes. Therefore, published data still do not permit a conclusive evaluation of the impact of soy IF on the methylome. Future studies will have to address this question in more detail, especially since several *in vivo* studies with focus on developmental impact of soy intervention suggest that *in utero* or perinatal exposure might influence DNA methylation, resulting in long-term reprogramming of gene expression.

Summary of the influence of IF on epigenetic “writers” (DNMTs, HATs, EZH2), “readers” (MeCP, MBD2), “editors” (miRNAs), and “erasers” (HDACs, SIRT1) modulating the main epigenetic mechanisms (inner circle), genes targeted by these activities (middle circle), and the “hallmarks of cancer” affected by the resulting changes in gene expression (outer circle). Further details are given in the text.

With the technological advancement and increased affordability of next generation sequencing (NGS), research during the past few years has provided a wealth of information on the regulation of gene expression through *histone modifications* and the genetic and epigenetic defects that deregulate these processes during carcinogenesis [158]. The influence of IF on histone modifications seems to play a consistent role in epigenetic regulation of gene expression. There is convincing evidence that IF lead to up-regulation of HATs, including HAT1, p300, PCAF, CBP, resulting in increased histone acetylation at the promoters of several genes, for example the cell cycle regulators p16 and p21, TSGs (PTEN, BTG3), Wnt-inhibitors (e.g., APC, SOX7, WIF1, DKK1, SFRP1, SFRP2), epigenetic regulators (EZH2, SRC3, p300), estrogen receptors (ER-α, ER-β), and the DNA repair gene BRCA1. Concomitantly, GEN was shown to down-regulate the expression of HDACs such as the estrogen-regulated HDAC6. As a consequence, through enhanced acetylation of the chaperone HSP90 and subsequent dissociation and degradation of the AR, AR-mediated signaling was inhibited in prostate cancer cells. Similarly, down-regulation of SIRT1 was associated with up-regulation of the TSG PTEN, inhibition of downstream AKT signaling and induction of apoptosis.

Deposition of histone methylation marks at selected gene promoters modulated by IF treatment has been addressed in several studies, mainly by chromatin immunoprecipitation (ChIP) followed by PCR. Gene up-regulation was indeed associated with increased levels of activating histone methylation marks (H3K4me2, H3K4me3) at promoters regions (e.g., p16, p21, BTG3). Underlying effects of IF on the activity or expression of HMT or HDM are largely unexplored. As an exception, short exposure of Eker rats to GEN at PND 10–12 inactivated the HMT EZH2 by phosphorylation, leading to reduced levels of repressive H3K27me3 levels and *promotion* of benign uterine tumors. Targeting the HMT distinguished GEN-mediated activity from that of the xenoestrogen bisphenol A.

Mechanistically, there is a link between hormone-mediated signaling and histone modification due to the fact that hormone receptor co-activators and co-repressors are associated with histone modifying enzymes (recent review in [159]). Since IF act as phytoestrogens, their binding to the ER recruits these enzymes to estrogen-responsive genes and contributes to targeted epigenetic regulation of gene expression. Interestingly, ER-α expression itself is silenced in ER-negative breast cancer cells through epigenetic mechanisms. GEN-induced enrichment of activating histone acetylation marks at the ER promoter resulted in ER-α re-expression and re-sensitized ER-negative breast cancer cells to the activity of tamoxifen, a selective estrogen receptor modulator (SERM) with tissue-dependent anti-estrogenic activity.

Only recently, *miRNAs* were shown to have an influence on cancer development and progression. A single miRNAs can target a multitude of genes and regulates signaling pathways often in a tissue-specific manner. Up- and down-regulation of several miRNAs were demonstrated to be excellent markers for diagnosis and prognosis of cancers, and miRNA profiling may be a useful tool for personalizing therapies. Within the past few years, numerous studies have indicated that treatment of cultured cells with soy IF modulates miRNA expression, for example of the miR-200 and let-7 family, miR-21, miR-34a, and miR-221/222. As a consequence, important cancer-related pathways are regulated, including Wnt- and Notch signaling, EMT associated genes (e.g., ZEB1/2, vimentin) and PTEN, contributing to IF-mediated inhibition of proliferation, invasion, migration and induction of apoptosis. Still, most of the studies were performed *in vitro*, and often, concentrations required to achieve these activities might be beyond the levels that can be reached through a diet rich in soy products. In the future, *in vivo* studies will have to prove that IF-induced miRNA-regulated effects are relevant for cancer prevention in animal models or even in humans. Also, the mechanisms underlying the influence of IF on miRNA expression will have to be elucidated.

With few exceptions, studies on the impact of IF on the epigenome focused on single epigenetic mechanism. However, a comparison of the data (see Figure 3) revealed that several genes or groups of genes that are epigenetically controlled at various levels can be targeted by IF by multiple mechanisms. Examples include the TSG PTEN, SFRPs and other inhibitors of Wnt-signaling, and cell cycle regulator p16. It remains to be analyzed whether these are exceptions or whether this is a general observation that has not been investigated in sufficient detail for other genes.

Does the impact of IF on epigenetic mechanisms play a role for cancer prevention? Epidemiological observations and the experimental studies summarized in this review suggest an influence, but definite proof of causal interactions in animal models or in humans is still missing. Technologies are now available to explore epigenetic mechanisms in rodent models for carcinogenesis at a genome-wide level, for example by RRBS and ChIP-NGS, and to relate them to binding of ER or other TF to their respective response elements, gene expression, and tumor growth. Taken together, these integrated datasets will help to fully understand the link between epigenetic gene regulation and cancer prevention by IF.

## References

[1] Stewart B.W., Wild C.P., IARC (2014). World Cancer Report 2014.

[2] World Cancer Research Fund/American Institute for Cancer Research (2007). Food, Nutrition, Physical Activity, and the Prevention of Cancer: A Global Perspective.

[3] Bingham S.A., Atkinson C., Liggins J., Bluck L., Coward A. (1998). Phyto-oestrogens: Where are we now?. Br. J. Nutr..

[4] Chen Z., Zheng W., Custer L.J., Dai Q., Shu X.O., Jin F., Franke A.A. (1999). Usual dietary consumption of soy foods and its correlation with the excretion rate of isoflavonoids in overnight urine samples among Chinese women in Shanghai. Nutr. Cancer.

[5] Seow A., Shi C.Y., Franke A.A., Hankin J.H., Lee H.P., Yu M.C. (1998). Isoflavonoid levels in spot urine are associated with frequency of dietary soy intake in a population-based sample of middle-aged and older Chinese in Singapore. Cancer Epidemiol. Biomark. Prev..

[6] Cassidy A., Faughnan M. (2000). Phyto-oestrogens through the life cycle. Proc. Nutr. Soc..

[7] Keinan-Boker L., Peeters P., Mulligan A., Navarro C., Slimani N., Mattisson I., Lundin E., McTaggart A., Allen N., Overvad K. (2002). Soy product consumption in 10 European countries: The European Prospective Investigation into Cancer and Nutrition (EPIC) study. Public Health Nutr..

[8] Clarke D.B., Barnes K.A., Castle L., Rose M., Wilson L.A., Baxter M.J., Price K.R., DuPont M.S. (2003). Levels of phytoestrogens, inorganic trace-elements, natural toxicants and nitrate in vegetarian duplicate diets. Food Chem..

[9] UK Ministry of Agriculture Fisheries and Food (MAFF UK) (1998). Plant oestrogens in soya-based infant formulae. Food Surveill. Inf. Sheet.

[10] Steiner C., Arnould S., Scalbert A., Manach C. (2008). Isoflavones and the prevention of breast and prostate cancer: New perspectives opened by nutrigenomics. Br. J. Nutr..

[11] Ferlay J., Soerjomataram I., Ervik M., Dikshit R., Eser S., Mathers C., Rebelo M., Parkin D.M., Forman D., Bray F. GLOBOCAN 2012 v1.0, Cancer Incidence and Mortality Worldwide: IARC CancerBase No. 11. http://globocan.iarc.fr.

[12] Ziegler R.G., Hoover R.N., Pike M.C., Hildesheim A., Nomura A.M.Y., West D.W., Wu-Williams A.H., Kolonel L.N., Horn-Ross P.L., Rosenthal J.F. (1993). Migration Patterns and Breast Cancer Risk in Asian-American Women. J. Natl. Cancer Inst..

[13] Tham D.M., Gardner C.D., Haskell W.L. (1998). Potential Health Benefits of Dietary Phytoestrogens: A Review of the Clinical, Epidemiological, and Mechanistic Evidence. J. Clin. Endocrinol. Metab..

[14] Mori M., Masumori N., Fukuta F., Nagata Y., Sonoda T., Sakauchi F., Ohnishi H., Nojima M., Tsukamoto T. (2009). Traditional Japanese diet and prostate cancer. Mol. Nutr. Food Res..

[15] Warri A., Saarinen N.M., Makela S., Hilakivi-Clarke L. (2008). The role of early life genistein exposures in modifying breast cancer risk. Br. J. Cancer.

[16] De Assis S., Hilakivi-Clarke L. (2006). Timing of Dietary Estrogenic Exposures and Breast Cancer Risk. Ann. N. Y. Acad. Sci..

[17] Hilakivi-Clarke L. (2007). Nutritional modulation of terminal end buds: Its relevance to breast cancer prevention. Curr. Cancer Drug Target.

[18] Hervouet E., Cartron P.F., Jouvenot M., Delage-Mourroux R. (2013). Epigenetic regulation of estrogen signaling in breast cancer. Epigenetics.

[19] Fang H., Tong W., Shi L.M., Blair R., Perkins R., Branham W., Hass B.S., Xie Q., Dial S.L., Moland C.L. (2001). Structure—Activity Relationships for a Large Diverse Set of Natural, Synthetic, and Environmental Estrogens. Chem. Res. Toxicol..

[20] Jones P.A., Baylin S.B. (2007). The Epigenomics of Cancer. Cell.

[21] Shen H., Laird P.W. (2013). Interplay between the cancer genome and epigenome. Cell.

[22] Greathouse K.L., Bredfeldt T., Everitt J.I., Lin K., Berry T., Kannan K., Mittelstadt M.L., Ho S.M., Walker C.L. (2012). Environmental estrogens differentially engage the histone methyltransferase EZH2 to increase risk of uterine tumorigenesis. Mol. Cancer Res..

[23] Esteller M. (2007). Cancer epigenomics: DNA methylomes and histone-modification maps. Nat. Rev. Genet..

[24] Santos F., Hendrich B., Reik W., Dean W. (2002). Dynamic reprogramming of DNA methylation in the early mouse embryo. Dev. Biol..

[25] Goelz S.E., Vogelstein B., Hamilton S.R., Feinberg A.P. (1985). Hypomethylation of DNA from benign and malignant human colon neoplasms. Science.

[26] Gama-Sosa M.A., Slagel V.A., Trewyn R.W., Oxenhandler R., Kuo K.C., Gehrke C.W., Ehrlich M. (1983). The 5-methylcytosine content of DNA from human tumors. Nucleic Acids Res..

[27] Stefanska B., Suderman M., Machnes Z., Bhattacharyya B., Hallett M., Szyf M. (2013). Transcription onset of genes critical in liver carcinogenesis is epigenetically regulated by methylated DNA-binding protein MBD2. Carcinogenesis.

[28] Mayol G., Martín-Subero J., Ríos J., Queiros A., Kulis M., Suñol M., Esteller M., Gómez S., Garcia I., de Torres C. (2012). DNA hypomethylation affects cancer-related biological functions and genes relevant in neuroblastoma pathogenesis. PLoS One.

[29] Stefanska B., Huang J., Bhattacharyya B., Suderman M., Hallett M., Han Z.-G., Szyf M. (2011). Definition of the Landscape of Promoter DNA Hypomethylation in Liver Cancer. Cancer Res..

[30] Pakneshan P., Szyf M., Rabbani S.A. (2005). Hypomethylation of Urokinase (uPA) Promoter in Breast and Prostate Cancer: Prognostic and Therapeutic Implications. Curr. Cancer Drug Targets.

[31] Rauch T.A., Wu X., Zhong X., Riggs A.D., Pfeifer G.P. (2009). A human B cell methylome at 100-base pair resolution. Proc. Natl. Acad. Sci. USA.

[32] Sato N., Fukushima N., Matsubayashi H., Goggins M. (2003). Identification of maspin and S100P as novel hypomethylation targets in pancreatic cancer using global gene expression profiling. Oncogene.

[33] Kopelovich L., Crowell J.A., Fay J.R. (2003). The epigenome as a target for cancer chemoprevention. J. Natl. Cancer Inst..

[34] Kouzarides T. (2007). Chromatin modifications and their function. Cell.

[35] Fullgrabe J., Kavanagh E., Joseph B. (2011). Histone onco-modifications. Oncogene.

[36] Sauve A.A., Wolberger C., Schramm V.L., Boeke J.D. (2006). The biochemistry of sirtuins. Annu. Rev. Biochem..

[37] Upadhyay A.K., Cheng X. (2011). Dynamics of histone lysine methylation: Structures of methyl writers and erasers. Prog. Drug Res..

[38] Laugesen A., Helin K. (2014). Chromatin Repressive Complexes in Stem Cells, Development, and Cancer. Cell Stem Cell.

[39] Bannister A.J., Kouzarides T. (2011). Regulation of chromatin by histone modifications. Cell Res..

[40] miRBase: The microRNA Database. http://mirbase.org.

[41] Winter J., Jung S., Keller S., Gregory R.I., Diederichs S. (2009). Many roads to maturity: MicroRNA biogenesis pathways and their regulation. Nat. Cell Biol..

[42] Calin G.A., Dumitru C.D., Shimizu M., Bichi R., Zupo S., Noch E., Aldler H., Rattan S., Keating M., Rai K. (2002). Frequent deletions and down-regulation of micro-RNA genes miR15 and miR16 at 13q14 in chronic lymphocytic leukemia. Proc. Natl. Acad. Sci. USA.

[43] Calin G.A., Croce C.M. (2006). MicroRNA signatures in human cancers. Nat. Rev. Cancer.

[44] Gupta R.A., Shah N., Wang K.C., Kim J., Horlings H.M., Wong D.J., Tsai M.C., Hung T., Argani P., Rinn J.L. (2010). Long non-coding RNA HOTAIR reprograms chromatin state to promote cancer metastasis. Nature.

[45] Prensner J.R., Iyer M.K., Sahu A., Asangani I.A., Cao Q., Patel L., Vergara I.A., Davicioni E., Erho N., Ghadessi M. (2013). The long noncoding RNA SChLAP1 promotes aggressive prostate cancer and antagonizes the SWI/SNF complex. Nat. Genet..

[46] Gutschner T., Hammerle M., Diederichs S. (2013). MALAT1—A paradigm for long noncoding RNA function in cancer. J. Mol. Med. (Berl.).

[47] Rietjens I.M.C.M., Sotoca A.M., Vervoort J., Louisse J. (2013). Mechanisms underlying the dualistic mode of action of major soy isoflavones in relation to cell proliferation and cancer risks. Mol. Nutr. Food Res..

[48] Fang M., Chen D., Yang C.S. (2007). Dietary Polyphenols May Affect DNA Methylation. J. Nutr..

[49] Grayson M. (2012). Breast cancer. Nature.

[50] National Cancer Institute (Bethesda, MD, USA): SEER Cancer Statistics Factsheets: All Cancer Sites. http://seer.cancer.gov/statfacts/html/all.html.

[51] Yager J.D., Davidson N.E. (2006). Estrogen Carcinogenesis in Breast Cancer. N. Engl. J. Med..

[52] Maxmen A. (2012). The hard facts. Nature.

[53] Nagata C., Mizoue T., Tanaka K., Tsuji I., Tamakoshi A., Matsuo K., Wakai K., Inoue M., Tsugane S., Sasazuki S. (2014). Soy Intake and Breast Cancer Risk: An Evaluation Based on a Systematic Review of Epidemiologic Evidence Among the Japanese Population. Jpn. J. Clin. Oncol..

[54] Messina M. (2010). A Brief Historical Overview of the Past Two Decades of Soy and Isoflavone Research. J. Nutr..

[55] Taylor C.K., Levy R.M., Elliott J.C., Burnett B.P. (2009). The effect of genistein aglycone on cancer and cancer risk: A review of *in vitro*, preclinical, and clinical studies. Nutr. Rev..

[56] Fritz H., Seely D., Flower G., Skidmore B., Fernandes R., Vadeboncoeur S., Kennedy D., Cooley K., Wong R., Sagar S. (2013). Soy, red clover, and isoflavones and breast cancer: A systematic review. PLoS One.

[57] Magee P.J., Rowland I. (2012). Soy products in the management of breast cancer. Curr. Opin. Clin. Nutr. Metab. Care.

[58] D’Adamo C.R., Sahin A. (2014). Soy foods and supplementation: A review of commonly perceived health benefits and risks. Altern. Ther. Health Med..

[59] Powles T.J. (2002). Anti-oestrogenic prevention of breast cancer—The make or break point. Nat. Rev. Cancer.

[60] Hong T., Nakagawa T., Pan W., Kim M.Y., Kraus W.L., Ikehara T., Yasui K., Aihara H., Takebe M., Muramatsu M. (2004). Isoflavones stimulate estrogen receptor-mediated core histone acetylation. Biochem. Biophys. Res. Commun..

[61] Jawaid K., Crane S.R., Nowers J.L., Lacey M., Whitehead S.A. (2010). Long-term genistein treatment of MCF-7 cells decreases acetylated histone 3 expression and alters growth responses to mitogens and histone deacetylase inhibitors. J. Steroid Biochem. Mol. Biol..

[62] Li Y., Meeran S.M., Patel S.N., Chen H., Hardy T.M., Tollefsbol T.O. (2013). Epigenetic reactivation of estrogen receptor-alpha (ERalpha) by genistein enhances hormonal therapy sensitivity in ERalpha-negative breast cancer. Mol. Cancer.

[63] Li Y., Chen H., Hardy T.M., Tollefsbol T.O. (2013). Epigenetic regulation of multiple tumor-related genes leads to suppression of breast tumorigenesis by dietary genistein. PLoS One.

[64] Dagdemir A., Durif J., Ngollo M., Bignon Y.J., Bernard-Gallon D. (2013). Histone lysine trimethylation or acetylation can be modulated by phytoestrogen, estrogen or anti-HDAC in breast cancer cell lines. Epigenomics.

[65] King-Batoon A., Leszczynska J.M., Klein C.B. (2008). Modulation of gene methylation by genistein or lycopene in breast cancer cells. Environ. Mol. Mutagen..

[66] Li Y., Liu L., Andrews L.G., Tollefsbol T.O. (2009). Genistein depletes telomerase activity through cross-talk between genetic and epigenetic mechanisms. Int. J. Cancer.

[67] Bosviel R., Dumollard E., Dechelotte P., Bignon Y.J., Bernard-Gallon D. (2012). Can soy phytoestrogens decrease DNA methylation in BRCA1 and BRCA2 oncosuppressor genes in breast cancer?. OMICS.

[68] Bosviel R., Durif J., Dechelotte P., Bignon Y.J., Bernard-Gallon D. (2012). Epigenetic modulation of BRCA1 and BRCA2 gene expression by equol in breast cancer cell lines. Br. J. Nutr..

[69] Xie Q., Bai Q., Zou L.-Y., Zhang Q.-Y., Zhou Y., Chang H., Yi L., Zhu J.-D., Mi M.-T. (2014). Genistein inhibits DNA methylation and increases expression of tumor suppressor genes in human breast cancer cells. Genes Chromosomes Cancer.

[70] Qin W., Zhu W., Shi H., Hewett J.E., Ruhlen R.L., MacDonald R.S., Rottinghaus G.E., Chen Y.C., Sauter E.R. (2009). Soy isoflavones have an antiestrogenic effect and alter mammary promoter hypermethylation in healthy premenopausal women. Nutr. Cancer.

[71] Sonnet M., Baer C., Rehli M., Weichenhan D., Plass C. (2013). Enrichment of methylated DNA by methyl-CpG immunoprecipitation. Methods Mol. Biol..

[72] Gu H., Bock C., Mikkelsen T.S., Jager N., Smith Z.D., Tomazou E., Gnirke A., Lander E.S., Meissner A. (2010). Genome-scale DNA methylation mapping of clinical samples at single-nucleotide resolution. Nat. Methods.

[73] Tang W.Y., Newbold R., Mardilovich K., Jefferson W., Cheng R.Y., Medvedovic M., Ho S.M. (2008). Persistent hypomethylation in the promoter of nucleosomal binding protein 1 (Nsbp1) correlates with overexpression of Nsbp1 in mouse uteri neonatally exposed to diethylstilbestrol or genistein. Endocrinology.

[74] Matsukura H., Aisaki K., Igarashi K., Matsushima Y., Kanno J., Muramatsu M., Sudo K., Sato N. (2011). Genistein promotes DNA demethylation of the steroidogenic factor 1 (SF-1) promoter in endometrial stromal cells. Biochem. Biophys. Res. Commun..

[75] Guerrero-Bosagna C.M., Sabat P., Valdovinos F.S., Valladares L.E., Clark S.J. (2008). Epigenetic and phenotypic changes result from a continuous pre and post natal dietary exposure to phytoestrogens in an experimental population of mice. BMC Physiol..

[76] Jha A.K., Nikbakht M., Parashar G., Shrivastava A., Capalash N., Kaur J. (2010). Reversal of hypermethylation and reactivation of the RARbeta2 gene by natural compounds in cervical cancer cell lines. Folia Biol. (Praha).

[77] Parker L.P., Taylor D.D., Kesterson J., Metzinger D.S., Gercel-Taylor C. (2009). Modulation of microRNA associated with ovarian cancer cells by genistein. Eur. J. Gynaecol. Oncol..

[78] Xu L., Xiang J., Shen J., Zou X., Zhai S., Yin Y., Li P., Wang X., Sun Q. (2013). Oncogenic MicroRNA-27a is a target for genistein in ovarian cancer cells. Anticancer Agents Med. Chem..

[79] Sun Q., Cong R., Yan H., Gu H., Zeng Y., Liu N., Chen J., Wang B. (2009). Genistein inhibits growth of human uveal melanoma cells and affects microRNA-27a and target gene expression. Oncol. Rep..

[80] Lyn-Cook B.D., Blann E., Payne P.W., Bo J., Sheehan D., Medlock K. (1995). Methylation profile and amplification of proto-oncogenes in rat pancreas induced with phytoestrogens. Proc. Soc. Exp. Biol. Med..

[81] Bosch F.X., Broker T.R., Forman D., Moscicki A.B., Gillison M.L., Doorbar J., Stern P.L., Stanley M., Arbyn M., Poljak M. (2013). Comprehensive control of human papillomavirus infections and related diseases. Vaccine.

[82] Karius T., Schnekenburger M., Dicato M., Diederich M. (2012). MicroRNAs in cancer management and their modulation by dietary agents. Biochem. Pharmacol..

[83] Costello A.J., Corcoran N.M., Tewari A. (2013). Development, applied and surgical development of the prostate. Prostate Cancer: A Comprehensive Perspective.

[84] Jemal A., Bray F., Center M.M., Ferlay J., Ward E., Forman D. (2011). Global cancer statistics. CA Cancer J. Clin..

[85] Kimura T. (2012). East meets West: Ethnic differences in prostate cancer epidemiology between East Asians and Caucasians. Chin. J. Cancer.

[86] Jian L. (2009). Soy, isoflavones, and prostate cancer. Mol. Nutr. Food Res..

[87] Basak S., Pookot D., Noonan E.J., Dahiya R. (2008). Genistein down-regulates androgen receptor by modulating HDAC6-Hsp90 chaperone function. Mol. Cancer Ther..

[88] Majid S., Kikuno N., Nelles J., Noonan E., Tanaka Y., Kawamoto K., Hirata H., Li L.C., Zhao H., Okino S.T. (2008). Genistein induces the p21WAF1/CIP1 and p16INK4a tumor suppressor genes in prostate cancer cells by epigenetic mechanisms involving active chromatin modification. Cancer Res..

[89] Kikuno N., Shiina H., Urakami S., Kawamoto K., Hirata H., Tanaka Y., Majid S., Igawa M., Dahiya R. (2008). Genistein mediated histone acetylation and demethylation activates tumor suppressor genes in prostate cancer cells. Int. J. Cancer.

[90] Majid S., Dar A.A., Shahryari V., Hirata H., Ahmad A., Saini S., Tanaka Y., Dahiya A.V., Dahiya R. (2010). Genistein reverses hypermethylation and induces active histone modifications in tumor suppressor gene B-Cell translocation gene 3 in prostate cancer. Cancer.

[91] Day J.K., Bauer A.M., DesBordes C., Zhuang Y., Kim B.E., Newton L.G., Nehra V., Forsee K.M., MacDonald R.S., Besch-Williford C. (2002). Genistein alters methylation patterns in mice. J. Nutr..

[92] Fang M.Z., Chen D., Sun Y., Jin Z., Christman J.K., Yang C.S. (2005). Reversal of hypermethylation and reactivation of p16INK4a, RARbeta, and MGMT genes by genistein and other isoflavones from soy. Clin. Cancer Res..

[93] Vardi A., Bosviel R., Rabiau N., Adjakly M., Satih S., Dechelotte P., Boiteux J.P., Fontana L., Bignon Y.J., Guy L. (2010). Soy Phytoestrogens Modify DNA Methylation of GSTP1, RASSF1A, EPH2 and BRCA1 Promoter in Prostate Cancer Cells. In Vivo.

[94] Adjakly M., Bosviel R., Rabiau N., Boiteux J.P., Bignon Y.J., Guy L., Bernard-Gallon D. (2011). DNA methylation and soy phytoestrogens: Quantitative study in DU-145 and PC-3 human prostate cancer cell lines. Epigenomics.

[95] Phillip C.J., Giardina C.K., Bilir B., Cutler D.J., Lai Y.H., Kucuk O., Moreno C.S. (2012). Genistein cooperates with the histone deacetylase inhibitor vorinostat to induce cell death in prostate cancer cells. BMC Cancer.

[96] Rabiau N., Trraf H.K., Adjakly M., Bosviel R., Guy L., Fontana L., Bignon Y.J., Bernard-Gallon D.J. (2011). miRNAs differentially expressed in prostate cancer cell lines after soy treatment. In Vivo.

[97] Zaman M.S., Chen Y., Deng G., Shahryari V., Suh S.O., Saini S., Majid S., Liu J., Khatri G., Tanaka Y. (2010). The functional significance of microRNA-145 in prostate cancer. Br. J. Cancer.

[98] Majid S., Dar A.A., Saini S., Chen Y., Shahryari V., Liu J., Zaman M.S., Hirata H., Yamamura S., Ueno K. (2010). Regulation of minichromosome maintenance gene family by microRNA-1296 and genistein in prostate cancer. Cancer Res..

[99] Chen Y., Zaman M.S., Deng G., Majid S., Saini S., Liu J., Tanaka Y., Dahiya R. (2011). MicroRNAs 221/222 and genistein-mediated regulation of ARHI tumor suppressor gene in prostate cancer. Cancer Prev. Res. (Phila.).

[100] Chiyomaru T., Yamamura S., Zaman M.S., Majid S., Deng G., Shahryari V., Saini S., Hirata H., Ueno K., Chang I. (2012). Genistein suppresses prostate cancer growth through inhibition of oncogenic microRNA-151. PLoS One.

[101] Li Y., Kong D., Ahmad A., Bao B., Dyson G., Sarkar F.H. (2012). Epigenetic deregulation of miR-29a and miR-1256 by isoflavone contributes to the inhibition of prostate cancer cell growth and invasion. Epigenetics.

[102] Chiyomaru T., Yamamura S., Fukuhara S., Hidaka H., Majid S., Saini S., Arora S., Deng G., Shahryari V., Chang I. (2013). Genistein up-regulates tumor suppressor microRNA-574-3p in prostate cancer. PLoS One.

[103] Chiyomaru T., Yamamura S., Fukuhara S., Yoshino H., Kinoshita T., Majid S., Saini S., Chang I., Tanaka Y., Enokida H. (2013). Genistein inhibits prostate cancer cell growth by targeting miR-34a and oncogenic HOTAIR. PLoS One.

[104] Hirata H., Hinoda Y., Shahryari V., Deng G., Tanaka Y., Tabatabai Z.L., Dahiya R. (2014). Genistein downregulates onco-miR-1260b and upregulates sFRP1 and Smad4 via demethylation and histone modification in prostate cancer cells. Br. J. Cancer.

[105] Majid S., Dar A.A., Ahmad A.E., Hirata H., Kawakami K., Shahryari V., Saini S., Tanaka Y., Dahiya A.V., Khatri G. (2009). BTG3 tumor suppressor gene promoter demethylation, histone modification and cell cycle arrest by genistein in renal cancer. Carcinogenesis.

[106] Zaman M.S., Shahryari V., Deng G., Thamminana S., Saini S., Majid S., Chang I., Hirata H., Ueno K., Yamamura S. (2012). Up-regulation of microRNA-21 correlates with lower kidney cancer survival. PLoS One.

[107] Zaman M.S., Thamminana S., Shahryari V., Chiyomaru T., Deng G., Saini S., Majid S., Fukuhara S., Chang I., Arora S. (2012). Inhibition of PTEN gene expression by oncogenic miR-23b-3p in renal cancer. PLoS One.

[108] Hirata H., Ueno K., Nakajima K., Tabatabai Z.L., Hinoda Y., Ishii N., Dahiya R. (2013). Genistein downregulates onco-miR-1260b and inhibits Wnt-signalling in renal cancer cells. Br. J. Cancer.

[109] Chiyomaru T., Fukuhara S., Saini S., Majid S., Deng G., Shahryari V., Chang I., Tanaka Y., Enokida H., Nakagawa M. (2014). Long non-coding RNA HOTAIR is targeted and regulated by miR-141 in human cancer cells. J. Biol. Chem..

[110] Weischenfeldt J., Simon R., Feuerbach L., Schlangen K., Weichenhan D., Minner S., Wuttig D., Warnatz H.-J., Stehr H., Rausch T. (2013). Integrative Genomic Analyses Reveal an Androgen-Driven Somatic Alteration Landscape in Early-Onset Prostate Cancer. Cancer Cell.

[111] Weiss M., Plass C., Gerhauser C. (2014). Role of lncRNAs in prostate cancer development and progression. Biol. Chem..

[112] Vivanco I., Sawyers C.L. (2002). The phosphatidylinositol 3-Kinase AKT pathway in human cancer. Nat. Rev. Cancer.

[113] Croce C.M. (2009). Causes and consequences of microRNA dysregulation in cancer. Nat. Rev. Genet..

[114] Esteller M. (2011). Non-coding RNAs in human disease. Nat. Rev. Genet..

[115] Dalai I., Missiaglia E., Barbi S., Butturini G., Doglioni C., Falconi M., Scarpa A. (2007). Low expression of ARHI is associated with shorter progression-free survival in pancreatic endocrine tumors. Neoplasia.

[116] Tsai M.C., Manor O., Wan Y., Mosammaparast N., Wang J.K., Lan F., Shi Y., Segal E., Chang H.Y. (2010). Long noncoding RNA as modular scaffold of histone modification complexes. Science.

[117] Yang Y., Liu J., Li X., Li J.C. (2012). PCDH17 gene promoter demethylation and cell cycle arrest by genistein in gastric cancer. Histol. Histopathol..

[118] Spurling C.C., Suhl J.A., Boucher N., Nelson C.E., Rosenberg D.W., Giardina C. (2008). The short chain fatty acid butyrate induces promoter demethylation and reactivation of RARbeta2 in colon cancer cells. Nutr. Cancer.

[119] Wang Z., Chen H. (2010). Genistein increases gene expression by demethylation of WNT5a promoter in colon cancer cell line SW1116. Anticancer Res..

[120] Zhang Y., Chen H. (2011). Genistein attenuates WNT signaling by up-regulating sFRP2 in a human colon cancer cell line. Exp. Biol. Med. (Maywood).

[121] Wang H., Li Q., Chen H. (2012). Genistein affects histone modifications on Dickkopf-related protein 1 (DKK1) gene in SW480 human colon cancer cell line. PLoS One.

[122] Zhang Y., Li Q., Chen H. (2013). DNA methylation and histone modifications of Wnt genes by genistein during colon cancer development. Carcinogenesis.

[123] Groh I.A., Chen C., Luske C., Cartus A.T., Esselen M. (2013). Plant polyphenols and oxidative metabolites of the herbal alkenylbenzene methyleugenol suppress histone deacetylase activity in human colon carcinoma cells. J. Nutr. Metab..

[124] Li Y., VandenBoom T.G., Kong D., Wang Z., Ali S., Philip P.A., Sarkar F.H. (2009). Up-regulation of miR-200 and let-7 by natural agents leads to the reversal of epithelial-to-mesenchymal transition in gemcitabine-resistant pancreatic cancer cells. Cancer Res..

[125] Li Y., Vandenboom T.G., Wang Z., Kong D., Ali S., Philip P.A., Sarkar F.H. (2010). miR-146a suppresses invasion of pancreatic cancer cells. Cancer Res..

[126] Xia J., Duan Q., Ahmad A., Bao B., Banerjee S., Shi Y., Ma J., Geng J., Chen Z., Rahman K.M. (2012). Genistein inhibits cell growth and induces apoptosis through up-regulation of miR-34a in pancreatic cancer cells. Curr. Drug Targets.

[127] Ma J., Cheng L., Liu H., Zhang J., Shi Y., Zeng F., Miele L., Sarkar F.H., Xia J., Wang Z. (2013). Genistein down-regulates miR-223 expression in pancreatic cancer cells. Curr. Drug Targets.

[128] Xia J., Cheng L., Mei C., Ma J., Shi Y., Zeng F., Wang Z., Wang Z. (2014). Genistein Inhibits Cell Growth and Invasion Through Regulation of MiR-27a in Pancreatic Cancer Cells. Curr. Pharm. Des..

[129] MacDonald B.T., Tamai K., He X. (2009). Wnt/β-Catenin Signaling: Components, Mechanisms, and Diseases. Dev. Cell.

[130] Bo H., Zhang S., Gao L., Chen Y., Zhang J., Chang X., Zhu M. (2013). Upregulation of Wnt5a promotes epithelial-to-mesenchymal transition and metastasis of pancreatic cancer cells. BMC Cancer.

[131] Cheng R., Sun B., Liu Z., Zhao X., Qi L., Li Y., Gu Q. (2014). Wnt5a Suppresses Colon Cancer by Inhibiting Cell Proliferation and Epithelial–Mesenchymal Transition. J. Cell. Physiol..

[132] Zhang Y., Li Q., Zhou D., Chen H. (2013). Genistein, a soya isoflavone, prevents azoxymethane-induced up-regulation of WNT/beta-catenin signalling and reduces colon pre-neoplasia in rats. Br. J. Nutr..

[133] Kalluri R., Weinberg R.A. (2009). The basics of epithelial-mesenchymal transition. J. Clin. Investig..

[134] Thiery J.P. (2002). Epithelial-mesenchymal transitions in tumour progression. Nat. Rev. Cancer.

[135] Yang J., Weinberg R.A. (2008). Epithelial-mesenchymal transition: At the crossroads of development and tumor metastasis. Dev. Cell.

[136] Gregory P.A., Bert A.G., Paterson E.L., Barry S.C., Tsykin A., Farshid G., Vadas M.A., Khew-Goodall Y., Goodall G.J. (2008). The miR-200 family and miR-205 regulate epithelial to mesenchymal transition by targeting ZEB1 and SIP1. Nat. Cell Biol..

[137] Park S.M., Gaur A.B., Lengyel E., Peter M.E. (2008). The miR-200 family determines the epithelial phenotype of cancer cells by targeting the *E*-cadherin repressors ZEB1 and ZEB2. Genes Dev..

[138] Zhang J., Ma L. (2012). MicroRNA control of epithelial-mesenchymal transition and metastasis. Cancer Metastasis Rev..

[139] Raynal N.J.M., Charbonneau M., Momparler L.F., Momparler R.L. (2008). Synergistic Effect of 5-Aza-2’-Deoxycytidine and Genistein in Combination Against Leukemia. Oncol. Res. Featur. Preclin. Clin. Cancer Ther..

[140] Li H., Xu W., Huang Y., Huang X., Xu L., Lv Z. (2012). Genistein demethylates the promoter of CHD5 and inhibits neuroblastoma growth *in vivo*. Int. J. Mol. Med..

[141] Dolinoy D.C., Weidman J.R., Waterland R.A., Jirtle R.L. (2006). Maternal genistein alters coat color and protects Avy mouse offspring from obesity by modifying the fetal epigenome. Environ. Health Perspect..

[142] Badger T.M., Ronis M.J., Wolff G., Stanley S., Ferguson M., Shankar K., Simpson P., Jo C.H. (2008). Soy protein isolate reduces hepatosteatosis in yellow Avy/a mice without altering coat color phenotype. Exp. Biol. Med. (Maywood).

[143] Rosenfeld C.S., Sieli P.T., Warzak D.A., Ellersieck M.R., Pennington K.A., Roberts R.M. (2013). Maternal exposure to bisphenol A and genistein has minimal effect on Avy/a offspring coat color but favors birth of agouti over nonagouti mice. Proc. Natl. Acad. Sci. USA.

[144] Sato N., Yamakawa N., Masuda M., Sudo K., Hatada I., Muramatsu M. (2011). Genome-wide DNA methylation analysis reveals phytoestrogen modification of promoter methylation patterns during embryonic stem cell differentiation. PLoS One.

[145] Vanhees K., Coort S., Ruijters E.J., Godschalk R.W., van Schooten F.J., Barjesteh van Waalwijk van Doorn-Khosrovani S. (2011). Epigenetics: Prenatal exposure to genistein leaves a permanent signature on the hematopoietic lineage. FASEB J..

[146] Vandegehuchte M.B., Lemière F., Vanhaecke L., vanden Berghe W., Janssen C.R. (2010). Direct and transgenerational impact on Daphnia magna of chemicals with a known effect on DNA methylation. Comp. Biochem. Physiol. C Toxicol. Pharmacol..

[147] Howard T.D., Ho S.M., Zhang L., Chen J., Cui W., Slager R., Gray S., Hawkins G.A., Medvedovic M., Wagner J.D. (2011). Epigenetic changes with dietary soy in cynomolgus monkeys. PLoS One.

[148] Scoccianti C., Ricceri F., Ferrari P., Cuenin C., Sacerdote C., Polidoro S., Jenab M., Hainaut P., Vineis P., Herceg Z. (2011). Methylation patterns in sentinel genes in peripheral blood cells of heavy smokers: Influence of cruciferous vegetables in an intervention study. Epigenetics.

[149] Bernal A.J., Jirtle R.L. (2010). Epigenomic disruption: The effects of early developmental exposures. Birth Defects Res. A Clin. Mol. Teratol..

[150] Brennan K., Garcia-Closas M., Orr N., Fletcher O., Jones M., Ashworth A., Swerdlow A., Thorne H., Investigators K.C., Riboli E. (2012). Intragenic ATM methylation in peripheral blood DNA as a biomarker of breast cancer risk. Cancer Res..

[151] Wang X., Zhu H., Snieder H., Su S., Munn D., Harshfield G., Maria B.L., Dong Y., Treiber F., Gutin B. (2010). Obesity related methylation changes in DNA of peripheral blood leukocytes. BMC Med..

[152] Houseman E.A., Accomando W.P., Koestler D.C., Christensen B.C., Marsit C.J., Nelson H.H., Wiencke J.K., Kelsey K.T. (2012). DNA methylation arrays as surrogate measures of cell mixture distribution. BMC Bioinform..

[153] Reinius L.E., Acevedo N., Joerink M., Pershagen G., Dahlen S.E., Greco D., Soderhall C., Scheynius A., Kere J. (2012). Differential DNA methylation in purified human blood cells: Implications for cell lineage and studies on disease susceptibility. PLoS One.

[154] Adalsteinsson B.T., Gudnason H., Aspelund T., Harris T.B., Launer L.J., Eiriksdottir G., Smith A.V., Gudnason V. (2012). Heterogeneity in white blood cells has potential to confound DNA methylation measurements. PLoS One.

[155] Hanahan D., Weinberg R.A. (2000). The hallmarks of cancer. Cell.

[156] Hanahan D., Weinberg R.A. (2011). Hallmarks of cancer: The next generation. Cell.

[157] Imielinski M., Berger A.H., Hammerman P.S., Hernandez B., Pugh T.J., Hodis E., Cho J., Suh J., Capelletti M., Sivachenko A. (2012). Mapping the hallmarks of lung adenocarcinoma with massively parallel sequencing. Cell.

[158] Plass C., Pfister S.M., Lindroth A.M., Bogatyrova O., Claus R., Lichter P. (2013). Mutations in regulators of the epigenome and their connections to global chromatin patterns in cancer. Nat. Rev. Genet..

[159] Romagnolo D.F., Zempleni J., Selmin O.I. (2014). Nuclear Receptors and Epigenetic Regulation: Opportunities for Nutritional Targeting and Disease Prevention. Adv. Nutr..

